# Biosynthesis of selenium nanoparticles with orange peel waste in microwave: characterization, and spectral properties

**DOI:** 10.1007/s13205-026-05115-0

**Published:** 2026-11-02

**Authors:** Tainá Pereira da Silva Oliveira, Alan Kelbis Oliveira Lima, Talita Pereira Gonçalves, Marcelo Henrique Sousa, José Antônio de Aquino Ribeiro, Clenilson Martins Rodrigues, Isadora Florêncio, Sônia Nair Báo, Dayane Batista Tada, Hugo de Campos Braga, Elton Clementino da Silva, Luis Alexandre Muehlmann

**Affiliations:** 1 Laboratory of Nanobiotechnology, Darcy Ribeiro University Campus, University of Brasília (UnB), Brasília, 70910-900 DF Brazil; 2 Brazilian Agricultural Research Corporation (EMBRAPA), Embrapa Agroenergy, Brasília, 70770- 901 DF Brazil; 3 Green Nanotechnology Group, University of Brasília (UnB), Brasília, 72220-900 DF Brazil; 4 Laboratory of Microscopy and Microanalysis, University of Brasília (UnB), Brasília, 70910-900 DF Brazil; 5 Laboratory of Nanomaterials and Nanotoxicology, Department of Science and Technology, Federal University of São Paulo, São José Dos Campos, SP Brazil; 6 School of Health Sciences and Technologies, Campus Ceilândia, University of Brasília (UnB), Brasília, 72220-275 Brazil

**Keywords:** Orange peel, SeNPs, Green synthesis, Electromagnetic radiation, Sodium selenite, Nanobiotechnology

## Abstract

The objective of this study was to reuse agroindustrial waste from orange peels to obtain an orange peel aqueous extract (OPEa), used in the biosynthesis and characterization of selenium nanoparticles (SeNPs). The OPEa acted as a reducing and stabilizing agent in the synthesis of SeNPs, due to its polyphenolic compounds, such as flavonoids, alkaloids, terpenoids, and coumarins, identified by UHPLC-HRMS/MS. The synthesis of SeNPs was conducted in a microwave reactor, at 120 °C and 150 °C, for 30 min, using different volumetric proportions/concentrations between OPEa and sodium selenite (Na₂SeO₃) (5 and 10 mM), in ratios of 1:4, 2:3, 1:1, 3:2, and 4:1 (OPEa_(mL)_:Na_2_SeO_3(mL)_). The SeNPs were characterized by visual inspection, UV/Vis, DLS, pH, TEM, SEM, and EDX. The results showed that SeNPs were successfully biosynthesized under all conditions, exhibiting distinct characteristics depending on the synthesis conditions. The formation of SeNPs was confirmed by the chromatic transition from greenish tones to light yellow to intense red and by UV/Vis spectrometry, with absorption peaks in the range of 250–340 nm. The SeNPs exhibited a hydrodynamic diameter (HD) of 213.6–1296 nm, with mono- and polydispersity characteristics (PdI: 0.033–0.885) and Zeta potential (ZP) between − 24.2 to -54.9 mV. Among the conditions tested, SeNPs with the best characteristics presented more homogeneous UV/Vis bands, lower HD (273.2–282.8 nm), and PdI (0.089–0.231), high ZP (-34.4 to -36.5 mV), stable pH (6), clear, translucent, and pellet-free colloidal solution, spherical morphology, and selenium accounted for 20.17% of its elemental composition.

**Supplementary Information:**

The online version contains supplementary material available at https://doi.org/10.1007/s13205-026-05115-0.

## Introduction

The orange (*Citrus spp.*) is one of the most important citrus crops worldwide, notable for its wide morphological and physicochemical versatility, as well as its high adaptability to different soil and climate conditions (Michael-Igolima et al. 2023; Ortiz-Sanchez et al. 2024). Global orange production is estimated at 60 million tons per year (Michael-Igolima et al. 2023), with Brazil accounting for around 15.7 million tons in 2024, with approximately 260 thousand orange trees cultivated, according to the Brazilian Institute of Geography and Statistics (IBGE 2024), of which only 50% are used for juice production, while the rest is discarded, making the country one of the largest generators of orange waste (Schneider et al. 2015; Pereira et al. 2022). Morphologically, the fruit is used mainly for its endocarp, the edible part formed by segments and juice vesicles, corresponding to 31–57% of its weight, while the remaining fraction (49–69% of its weight), which is inedible - the peel (epicarp and mesocarp) and seeds - is discarded and considered waste (Ayala et al. 2021; Ortiz-Sanchez et al. 2024).

Among the inedible parts of oranges, the peel poses the greatest challenge to the citrus industry. Although it is a rich source of antioxidants, polyphenols, pectin, fiber, essential oils, carotenoids, sugars, cellulose, and hemicellulose, with potential for biochemical applications such as bioethanol and biogas production (Ayala et al. 2021; Ortiz-Sanchez et al. 2024), its improper disposal, due to their high moisture content (80–90%), makes it susceptible to microbial degradation, cross-contamination, leachate production, and gas emissions (Gouveia 2023; Ortiz-Sanchez et al. 2024; Paini et al. 2025). Beyond its value as an agro-industrial waste, orange peel is particularly attractive for the green synthesis of selenium nanoparticles (SeNPs) due to its high content of polyphenols, flavonoids, and other redox-active phytochemicals, which act both as reducing agents for selenium ions and as stabilizing agents for the nanoparticles formed. Therefore, the green synthesis of SeNPs represents a sustainable strategy to mitigate the environmental impacts associated with orange peel disposal while simultaneously valorizing this agro-industrial waste (Aziz et al. 2022; Dang-Bao et al. 2022; Salem et al. 2022).

The selenium (from the Greek, σελήνη - “*Selene*”, moon) is a non-metallic element of the chalcogen group, belonging to the 16th group of the periodic table (Mikhailova 2023). Although it is considered an essential micronutrient, participating in fundamental biological processes such as immune, antioxidant, and metabolic functions, it can also be genotoxic depending on the dose, bioavailability and chemical form of exposure (organic - safer, or inorganic - more toxic) (Bjørklund et al. 2022; Genchi et al. 2023; Bhiri et al. 2025; Oliveira et al. 2025). Chemically, selenium is an element with high electronic versatility, capable of assuming multiple oxidation states (Bjørklund et al. 2022; Genchi et al. 2023; Bhiri et al. 2025), whose organic form has antioxidant, anticancer, antiviral and antimicrobial properties, while the inorganic form acts as genotoxic and participates in biological and biogeochemical cycles (Shamberger 1985; Pecoraro et al. 2022; Oliveira et al. 2025).

In contrast, SeNPs are more bioavailable, biocompatible, less toxic, and chemically more stable than selenium in its pure form, as well as metal nanoparticles such as silver, gold, copper, and magnesium, especially in biological applications (Khurana et al. 2019; Au et al. 2023; Oliveira et al. 2025; Satti et al. 2025). The synthesis of SeNPs depends on the strict control of several parameters that directly determine their morphological, physicochemical, optical, electronic, and magnetic properties. Among the most important factors are the nucleation and growth mechanisms involved in nanoparticles formation: LaMer nucleation (rapid nucleation followed by a separate growth stage); the Finke-Watzky mechanism (slow continuous nucleation associated with autocatalytic growth); Ostwald ripening (grow through the dissolution and redeposition of smaller particles); digestive ripening (formation of smaller and more uniform particles); coalescence by oriented attachment (fusion of smaller nanoparticles into larger structures through crystallographic alignment); and intraparticle growth (morphological changes driven by monomer redistribution within the nanoparticle). In addition, the synthesis route employed, whether chemical, physical, or biological, strongly influences nanoparticles characteristics. The final properties of SeNPs are also highly dependent on synthesis conditions, including the concentration, type, and proportion of reagents such as precursors, reducing agents, and stabilizers, as well as pH, temperature, and reaction time (Chaudhary et al. 2014; Kumar and Rajeshkumar 2018; Mellinas et al. 2019; Borowska et al. 2025; Oliveira et al. 2025).

Among the methods of synthesis, biosynthesis or green synthesis has stood out for its ability to bioreduce and stabilize selenium precursors by the biomolecules present in biological organisms, especially botanical species, such as flavonoids, alkaloids, and terpenoids, through their functional groups, such as hydroxyl (-OH), carboxyl (-COOH), and amino (-NH_2_) (Mellinas et al. 2019; Mikhailova 2023; Oliveira et al. 2025). Furthermore, biosynthesis has been improved using microwaves, which promote rapid and efficient heating of the solution of selenium ions and reducing agents, favoring biological organisms that require high temperatures during the process (Mellinas et al. 2019; Borowska et al. 2023; Borowska et al. 2025).

Considering this, the present study aimed to reutilize orange peels discarded by establishments in the Federal District, Brazil, through nanotechnological valorization by using an orange peel aqueous extract (OPEa) for the biosynthesis of SeNPs thereby promoting economic, environmental, sustainable, and social benefits. This study was based on the hypothesis that the biomolecules present in OPEa would act as reducing and stabilizing agents, enabling the synthesis of stable SeNPs, and that the combined effects of the sodium selenite (Na₂SeO₃) and OPEa ratio and microwave-assisted synthesis would significantly influence their physicochemical properties and long-term stability, thereby advancing the green synthesis of SeNPs through the valorization of agro-industrial waste. The main objectives included: (I) optimizing the synthesis of SeNPs using OPEa and Na₂SeO₃; (II) evaluating how the concentrations and ratios between Na₂SeO₃ and OPEa, and the use of a microwave reactor, influence the characteristics of SeNPs; (III) identifying the synthesis conditions that ensure the long-term stability of SeNPs; and (IV) characterizing the physicochemical properties of SeNPs over time.

## Materials and methods

### Materials and reagents

For the preparation of the orange peel aqueous extract (OPEa), orange peel residues were used. The synthesis of selenium nanoparticles (SeNPs) mediated by OPEa (SeNPs-OPEa) was carried out using sodium selenite (Na₂SeO₃) (Sigma-Aldrich, USA) and OPEa. For the characterization of OPEa, formic acid (CH₂O₂) (Sigma-Aldrich, USA) and acetonitrile (C₂H₃N) (Sigma-Aldrich, USA) of LC-MS analytical grade (> 98%), the free radical 2,2-diphenyl-1-picrylhydrazyl (DPPH) (Sigma-Aldrich, USA), methanol (CH_3_OH) (Dinâmica, Brazil), gallic acid (C_7_H_6_O_5_ - GAE) (Dinamica, Brazil), and *Folin-Ciocalteu* reagent (Dinamica, Brazil) were employed. All chemical reagents used in this study were used without additional purification.

### Preparation of OPEa

**Fig. 1:**
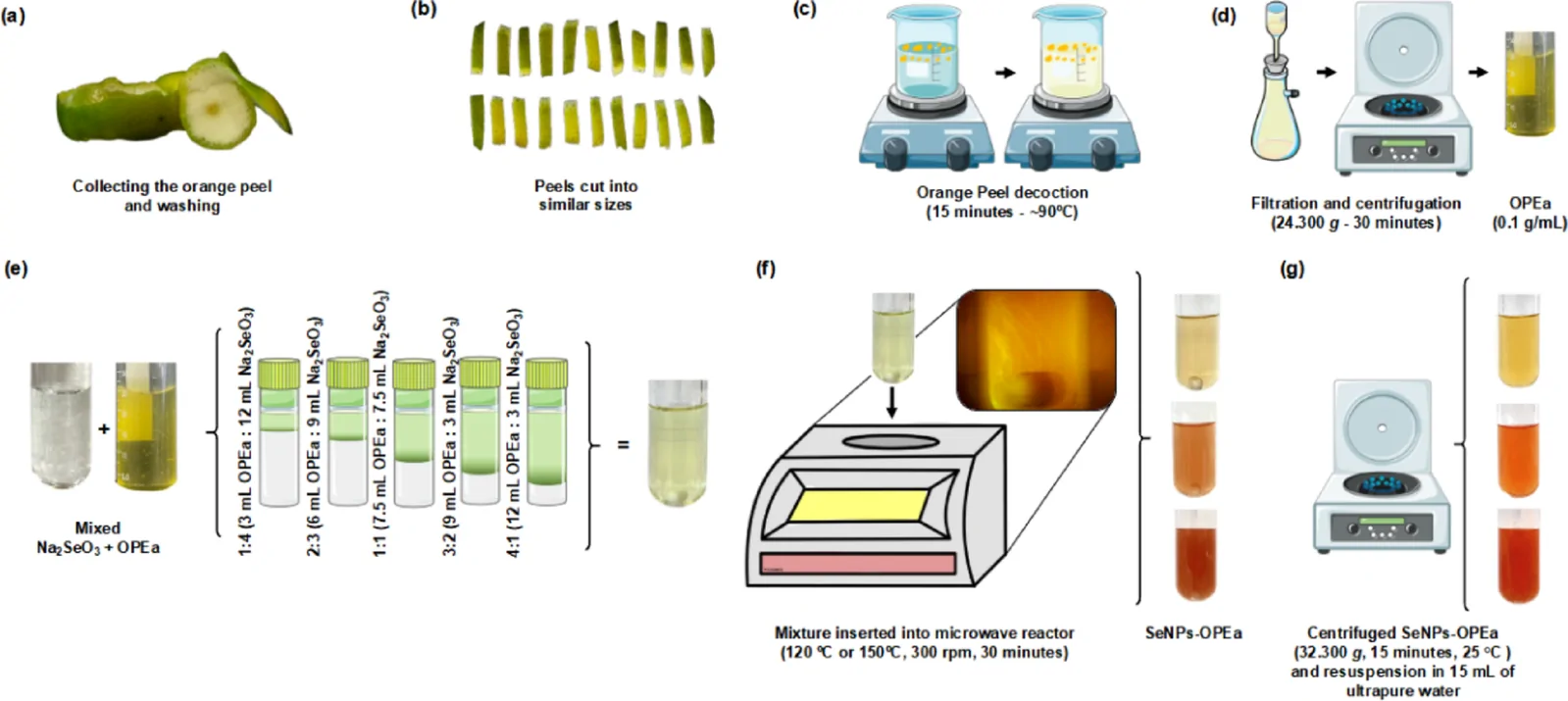
Diagram of the biosynthesis protocol for selenium nanoparticles (SeNPs) mediated by orange peel aqueous extract (OPEa) and sodium selenite (Na₂SeO₃) (SeNPs-OPEa) in a microwave reactor. The SeNPs-OPEa were synthesized at different temperatures (120 °C and 150 °C), without or with purification (WOP and WP, respectively), and at different volumetric ratios between OPEa and Na₂SeO₃ (OPEa_(mL)_ : Na₂SeO₃_(mL)_) − 1:4 (3 mL : 12 mL), 2:3 (6 mL : 9 mL), 1:1 (7.5 mL : 7.5 mL), 3:2 (9 mL : 6 mL) and 4:1 (12 mL : 3 mL). Preparation of OPEa (**a**-**d**); and Synthesis of SeNPs-OPEa under different microwave reactor conditions (**e**-**g**). The figure was designed using Images adapted from Servier Medical Art (https://smart.servier.com/*)*, licensed under CC BY 4.0 (https://creativecommons.org/licenses/by/4.0/)

The OPEa was obtained from orange peel waste, composed of the epicarp and mesocarp, collected from natural juice production facilities in Federal District, Brazil. To prepare the extract, the peels were initially washed in running water, followed by immersion in ultrapure water for 5 min to remove impurities and possible surface contaminants (Fig. 1a). The peels were then dried at room temperature with paper towels to remove excess moisture and then cut into similar sizes and shapes (Fig. 1b). The preparation followed the decoction method and was always performed immediately before use in the tests. After drying, the bark was added to boiling ultrapure water and kept boiling for 15 min (~ 90 °C) to obtain OPEa (Fig. 1c), which was subsequently filtered through qualitative filter paper (Whatman nº 1) and centrifuged at 24.300 *g* for 30 min (Hitachi CR22GIII) at 25 °C to remove tissue residues and purify the final OPEa (Fig. 1d). The volume of ultrapure water used for the preparation of OPEa followed a 1000:10 (m_(mg)_ : v_(mL)_) (peel : ultrapure water), reaching a final concentration of 100 mg/mL. The final coloration intensity of the OPEa varied in different shades of green, depending on the peels used.

### Biosynthesis of SeNPs-OPEa

To perform the biosynthesis of SeNPs-OPEa, aqueous solutions of Na₂SeO₃, at concentrations of 5 mM and 10 mM, were previously prepared and mixed with OPEa, obtained at a standard concentration of 100 mg/mL, in varying volumetric proportions. The mixtures were prepared in a G30 borosilicate glass reaction vessel (Anton Paar; operating capacity of 6–20 mL), adjusting the total volume to 15 mL, following the volumetric ratios between OPEa and Na₂SeO₃. The addition sequence consisted of introducing Na₂SeO₃, followed by OPEa (Fig. 1e). The different volumetric proportions of the mixtures resulted in distinct final concentrations, determined from the dilution of the stock solutions (Tables 1 and 2). After mixing the components, the reaction vessel was sealed with a pressure cap equipped with a silicone septum, ensuring complete sealing, and heated in a microwave reactor (Monowave 300 - Anton Paar) at reaction temperatures of 120–150 °C for 30 min (Fig. 1f). The microwave reactor was set to operate at 850 W power, 300 rpm magnetic stirring, and a cooling system maintained at 55 °C. After the reaction period ended, the SeNPs-OPEa colloidal solutions were transferred to plastic tubes for analysis without purification (WOP) and with purification (WP). For purification, the samples were centrifuged at 32.300 *g* for 15 min at 25 °C, the supernatant was discarded, and the pellet was resuspended with 15 mL of ultrapure water for analysis (Fig. 1g). After synthesis, the samples were stored under refrigeration (~ 4 °C) for seven days for characterization.

**Table 1:** Final concentrations of the aqueous extract of orange peel and 5 mM sodium selenite obtained from the different volumetric proportions used in the biosynthesis of selenium nanoparticles (SeNPs), calculated based on the stock concentrations, and the theoretical final concentration calculated for SeNPs

Samples (OPEa: Na₂SeO₃)	V_OPEa_:V_Na₂SeO₃_ (mL: mL)	FC_OPEa_ (mg/mL)	FC_Na₂SeO₃_5mM_ (mg/mL)	C_theor_SeNPs−OPEa_ (mg/mL)
1:4	3:12	20	0.692	0.316
2:3	6:9	40	0.519	0.237
1:1	7.5:7.5	50	0.433	0.198
3:2	9:6	60	0.346	0.158
4:1	12:3	80	0.173	0.079

Orange peel aqueous extract (OPEa); Sodium selenite (Na₂SeO₃); Selenium nanoparticles (SeNPs); Selenium nanoparticles mediated by OPEa (SeNPs-OPEa); Volume of OPEa used (V_OPEa_); Volume of Na₂SeO₃ used (V_Na₂SeO₃_); Final concentration of OPEa (FC_OPEa_); Final concentration of Na_2_SeO_3_ (FC_Na₂SeO₃_); and Theoretical concentration of SeNPs-OPEa (C_theor_SeNPs−OPEa_), assuming complete conversion of Na₂SeO₃ into elemental selenium (Se^0^). Stock concentrations: OPEa = 100 mg/mL; and 5 mM Na₂SeO₃ (0.865 mg/mL)

**Table 2:** Final concentrations of the aqueous extract of orange peel and 10 mM sodium selenite obtained from the different volumetric proportions used in the biosynthesis of selenium nanoparticles (SeNPs), calculated based on the stock concentrations, and the theoretical final concentration calculated for SeNPs

Samples (OPEa: Na_2_SeO_3_)	V_OPEa_:V_Na2SeO3_ (mL: mL)	FC_OPEa_ (mg/mL)	FC_Na2SeO3_10mM_ (mg/mL)	C_theor_SeNPs−OPEa_ (mg/mL)
1:4	3:12	20	1.38	0.632
2:3	6:9	40	1.04	0.474
1:1	7.5:7.5	50	0.866	0.396
3:2	9:6	60	0.692	0.316
4:1	12:3	80	0.346	0.158

Orange peel aqueous extract (OPEa); Sodium selenite (Na₂SeO₃); Selenium nanoparticles (SeNPs); Selenium nanoparticles mediated by OPEa (SeNPs-OPEa); Volume of OPEa used (V_OPEa_); Volume of Na₂SeO₃ used (V_Na₂SeO₃_); Final concentration of OPEa (FC_OPEa_); Final concentration of Na_2_SeO_3_ (FC_Na₂SeO₃_); and Theoretical concentration of SeNPs-OPEa (C_theor_SeNPs−OPEa_), assuming complete conversion of Na₂SeO₃ into elemental selenium (Se^0^). Stock concentrations: (1) OPEa: 100 mg/mL; and (2) Na₂SeO₃ at 10 mM or 1.73 mg/mL

### Characterization of OPEa: phytochemical and antioxidant activity

#### Ultra-high-performance liquid chromatography coupled to high-resolution mass spectrometry (UHPLC-HRMS/MS)

The phytochemical profile of the metabolites present in OPEa was determined by UHPLC-HRMS/MS, as in a previous study, with adaptations (Lima et al. 2025). Briefly, OPEa (100 mg/mL) was diluted to 5 mg/mL in ultrapure water, centrifuged at 12.400 *g* for 10 min, and subjected to chromatographic analysis on an Acquity UPLC HSST3 (100 × 2.1 mm, 1.8 μm) maintained at 40 °C, with a flow rate of 0.4 mL/min and an injection volume of 0.001 mL, using an elution gradient with formic acid in water (0.1% v: v) and formic acid in acetonitrile (0.1% v: v). The mass spectrometer was operated in positive and negative polarities (Auto MS/MS mode). Raw data were acquired in Compass 1.7 and processed in DataAnalysis 4.2 (Bruker Daltonics, Germany). First-order spectra (MS) were evaluated with SmartFormula and Compound Crawler, using the KEGG database as a basis, while second-order spectra (MS/MS) were evaluated with MetFrag.

#### Quantification of total phenol content (TPC)

The TPC of OPEa was determined by the *Folin-Ciocalteu* assay according to the previous protocol (Pires et al. 2017a). The OPEa assay followed the original protocol, with no changes in component volumes, while for the construction of the standard curve, the protocol was adapted, using six concentrations of gallic acid (C_7_H_6_O_5_ - GAE) (0.2 mg/mL) with volumes adjusted fourfold more for all components, totaling 1.2 mL in 24-well microplates (Kasvi, USA). The sequence of adding the components to the microplates during the tests followed the orders indicated in Table S1 and Table S2, respectively. Both tests were performed in triplicate from three independent experiments, with incubation of the microplates for 60 min in the dark at room temperature (~ 25 °C), with readings taken every 20 min and absorbance measured at 760 nm in a multimodal microplate reader (Varioskan LUX, Thermo Fisher Scientific) at 25 °C after the incubation period. The content of reducing substances was expressed in equivalents of the standard (gallic acid - C_7_H_6_O_5_ - GAE) per mass of plant extract (µg GAE/g OPEa).

#### Evaluation of the antioxidant activity of OPEa by the DPPH method

The antioxidant potential of OPEa was evaluated by the DPPH radical scavenging assay, according to the previous protocol (Pires et al. 2017b). Briefly, 0.28 mL of freshly prepared DPPH (0.1 mM) methanolic solution was mixed with 0.02 mL of OPEa (100 mg/mL) and the microplates were incubated for 30 and 60 min in the dark at room temperature (~ 25 °C). The standard curve assay was performed with adaptations, using five concentrations of gallic acid (C_7_H_6_O_5_ - GAE) (0.1 mg/mL), with volumes adjusted to four times more than all components used, adapted to a final volume of 1.2 mL in 24-well microplates (Kasvi, USA). The sequence of adding the components to the microplates during the assays followed the orders indicated in Table S3 and Table S4, respectively. Both assays were performed in triplicate from three independent experiments. After the incubation period, the analyses were conducted in a multimodal microplate reader (Varioskan LUX, Thermo Fisher Scientific), with absorbance readings at 517 nm and 25 °C. The content of reducing substances was expressed as gallic acid (C_7_H_6_O_5_ - GAE) equivalents per mass of OPEa (µg GAE/g OPEa).

### Characterization of SeNPs-OPEa

#### Ultraviolet/visible spectroscopy (UV/Vis)

The formation kinetics of SeNPs was analyzed using a UV/Vis spectrophotometer (HITACHI U-3900 H). To obtain the UV/Vis spectra, the samples were diluted at a ratio of 1:10 (v: v) (SeNPs-OPEa : ultrapure water), totaling a final volume of 2 mL. Spectrophotometric analyses were conducted over seven days, with measurements taken at intervals (days - D): D0 (day of synthesis), D1, and D7 (subsequent days). All analyses were performed at room temperature, including previously refrigerated samples. The equipment was configured for readings in the range of 200 to 600 nm, at 25 °C, using a quartz cuvette.

#### Dynamic light scattering (DLS) and zeta potential (ZP)

The SeNPs-OPEa were characterized in terms of hydrodynamic diameter (HD), polydispersity index (PdI), and Zeta potential (ZP) using ZetaSizer Nano ZS equipment (Malvern Instruments, UK) in polystyrene cuvettes. For analysis, the samples were diluted in ultrapure water at a ratio of 1:10 (v: v), obtaining a final volume of 1 mL. The samples were introduced into the equipment pre-configured for measurements with a helium-neon laser (633 nm) at 90°, with temperature stabilization at 25 °C for 30 s and 10 triplicate readings. The equipment settings and data processing were performed using ZetaSizer 7.13 software, developed by the equipment manufacturer. The colloidal stability of the samples was evaluated over seven days, with measurements taken at intervals (days - D): D0 (day of synthesis), D1, and D7 (subsequent days). All analyses were performed at room temperature, including previously refrigerated samples.

#### Transmission electron microscopy (TEM)

Morphological analysis and dry diameter measurement of SeNPs-OPEa were performed using a JEOL JEM-1011 transmission electron microscope (Tokyo, Japan), operated at 80 kV. For this, 3 µL of the pure colloidal solution were deposited onto 400 mesh copper grids coated with Formvar^®^ film and kept at room temperature in the dark for 24 h for complete drying. This equipment was available at the Laboratory of Microscopy and Microanalysis at the University of Brasília (UnB).

#### Scanning electron microscopy and energy-dispersive X-ray spectroscopy (SEM/EDX)

To obtain micrographs and determine the elemental composition and selenium content in SeNPs-OPEa, SEM and EDX were performed, respectively, using a scanning electron microscope (FEI, INSPECT S50, with detector Everhart-Thornley, Hillsboro, OR, EUA) operating at 10 kV available at NAPCEM Federal University of São Paulo. For these analyses, the SeNPs-OPEa were deposited on aluminum supports (stubs) without metallization and kept in the dark for 72 h to ensure complete drying prior to analysis.

### Statistical analysis

The statistical analyses of the parameters obtained by DLS (HD, PdI, and ZP) were performed using one-way analysis of variance (ANOVA), followed by Tukey’s multiple comparison test, considering a significance level of 95% (*p* < 0.05). All statistical analyses, as well as the construction of UV/Vis and DLS graphical representations, were conducted using GraphPad Prism 8 software (GraphPad Software Inc., San Diego, CA, USA).

## Results and discussion

### Redox-active phytochemical profile and antioxidant capacity of orange peel aqueous extract (OPEa)

Together, the results obtained by Ultra-High-Performance Liquid Chromatography coupled to High-Resolution Mass Spectrometry (UHPLC-HRMS/MS), Total Phenolic Content (TPC), and antioxidant activity (DPPH − 2,2-diphenyl-1-picrylhydrazyl) consistently indicate that OPEa retains redox-active phytochemicals capable of supporting the subsequent green synthesis of SeNPs, as detailed below.

#### UHPLC-HRMS/MS characterization

The characterization of phytochemicals present in OPEa was performed by UHPLC-HRMS/MS in positive (ESI(+)-MS) and negative (ESI(-)-MS) ionization modes, which assign charge to molecules, allowing the separation of ions by mass analyzers based on the mass-to-charge ratio (*m/z*) and the generation of high-resolution spectra, essential for the identification of compounds in the sample. The peaks, retention times (t_R_), mass-to-charge ratios (*m/z*) in positive and negative ionization modes, nomenclature, and class of the identified molecules are shown in Table 3.

**Table 3:**
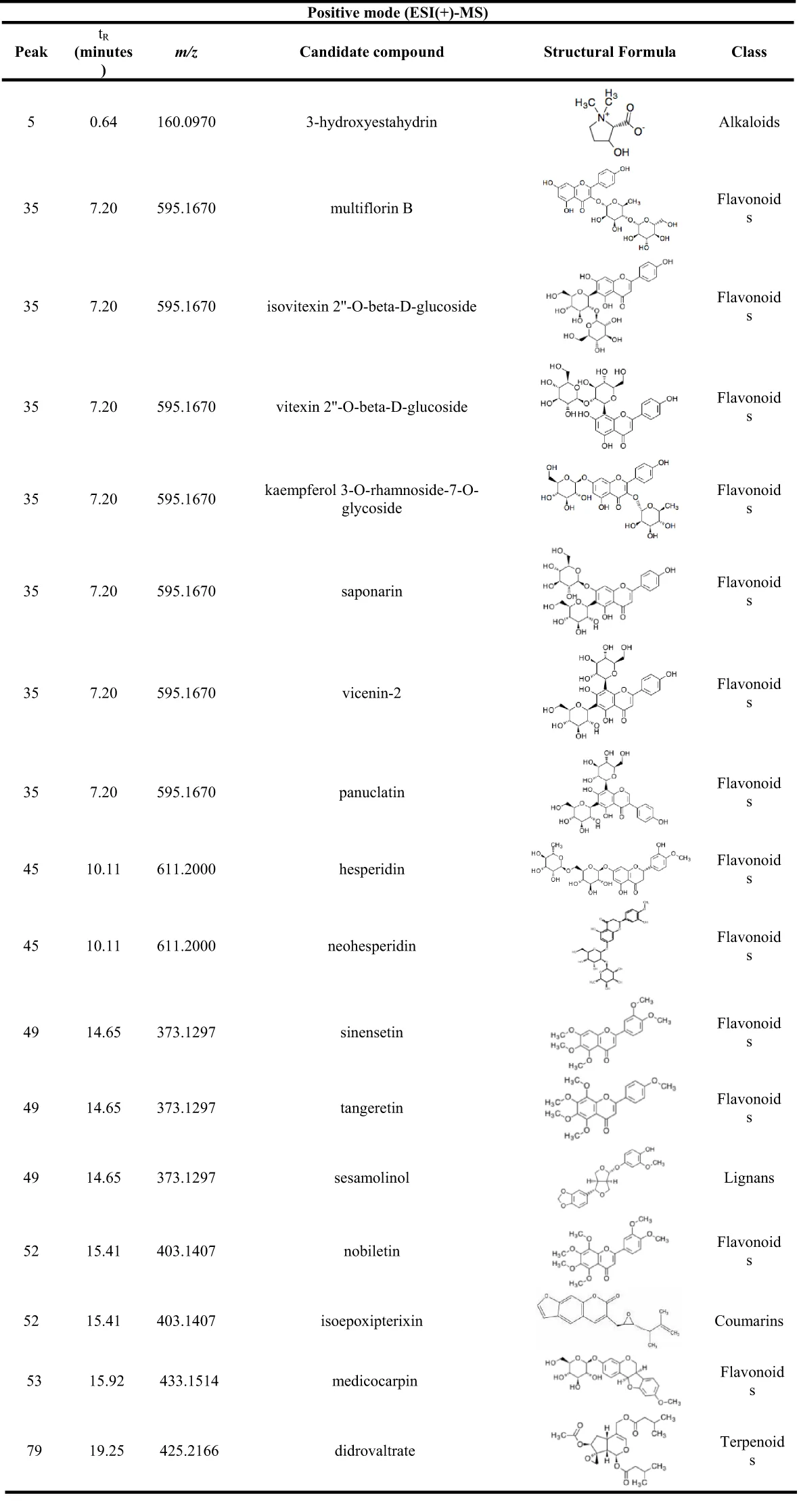

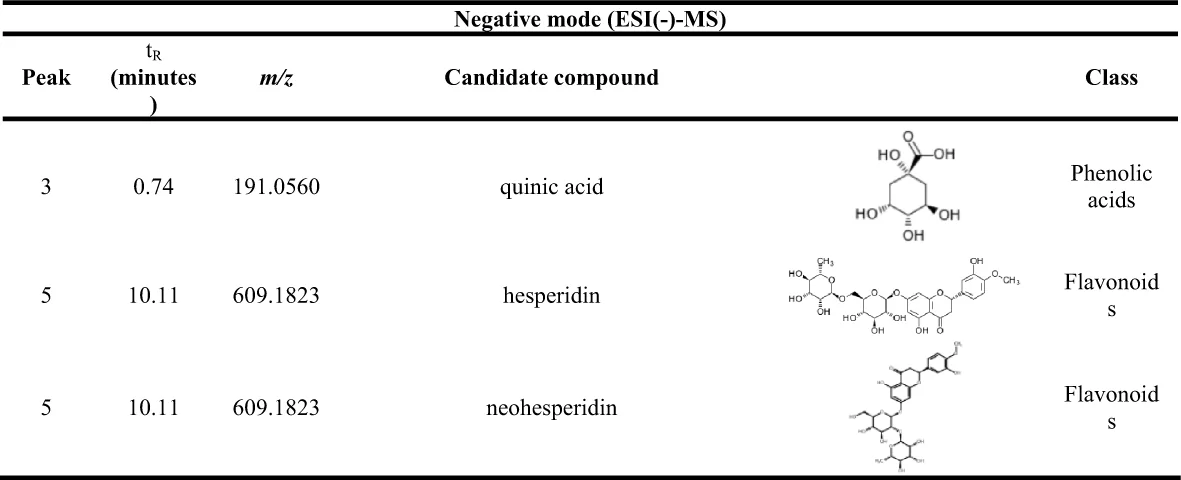
Compounds eluted from the orange peel aqueous extract (OPEa) and their respective mass-to-charge ratios (*m/z*) were identified through mass spectra obtained in positive (ESI(+)-MS) and negative (ESI(-)-MS) ionization modes

Retention time (t_R_); and mass-to-charge ratios (*m/z*)

Analysis of the base peak chromatogram (BPC) (Fig. S1) of OPEa detected eighteen biomolecules, mostly belonging to the flavonoid class, in addition to terpenoids, alkaloids, coumarins, and phenolic acids. Among the flavonoids, hesperidin and neohesperidin stood out for being present in both ionization modes, with protonated ions at *m/z* 611.2000 [M + H]^+^ (peak 45) and deprotonated ions at *m/z* 609.1823 [M-H]^-^ (peak 5), both with a retention time (t_R_) of 10.11 min. Among the terpenoids, only didrovaltrate was identified, making it the sole compound of this class, presenting protonated ions with *m/z* 425.2166 [M + H]^+^ (peak 79, t_R_ 19.25). The peaks 3 (negative mode), 5, 35, 49, 52, and 53 (positive mode) were also found in OPEa, representing the metabolites: quinic acid (191.0560; t_R_ 0.74); 3-hydroxyestahydrin (160.0970; t_R_ 0.65); multiflorin B, isovitexin 2’’-O-beta-D-glucoside, vitexin 2’’-O-beta-D-glucoside, kaempferol 3-O-rhamnoside-7-O-glycoside, saponarin, vicenin-2, panuclatin (595.1670; t_R_ 7.21); sinensetin, tangeretin, sesamolinol (373.1297; t_R_ 14.65); nobiletin, isoepoxipterixin (403.1407; t_R_ 15.41); a medicocarpin (433.1514; t_R_ 15.92), respectively.

The results obtained were consistent with data previously reported in the literature. Although the aqueous decoction extraction yielded fewer identified metabolites than organic-solvent extractions, likely due to the limited solubility of certain biomolecules in water and the characteristics of the extraction process (Castro-López et al. 2017; Rodrigues and Pintado 2024), the aqueous extract retained the classes of phytochemicals most frequently associated with nanoparticle reduction and stabilization. Therefore, the metabolites identified by UHPLC-HRMS/MS support the suitability of the aqueous extract for the green biosynthesis of selenium nanoparticles mediated by OPEa (SeNPs-OPEa). Moreover, the use of water as the extraction solvent is consistent with the principles of green chemistry, providing a safe, sustainable, and economically viable approach for the biosynthesis of SeNPs-OPEa and their biological applications.

Among the identified classes, flavonoids and terpenoids are commonly found in citrus fruits and play a key role in the synthesis of SeNPs by reducing selenite ions to elemental selenium (Se^0^). This reduction is mainly mediated by hydroxyl groups (-OH) present in these phytochemicals, which donate electrons during the reaction. In addition, these compounds contribute to the stabilization of the nanostructures by acting as coating agents (Menon et al. 2020; Pyrzynska and Sentkowska 2022; Shahzamani et al. 2022; Venkata et al. 2022). Among the compounds identified, the glycosylated flavonoids (neohesperidin, hesperidin, and vicenin-2) and the polymethoxylated flavonoids (tangeretin, nobiletin, and sinensetin) are the most widely reported due to their abundant presence in citrus fruit extracts, especially in the peel, compared to the juice or seeds, and for their antioxidant, anticancer, antiviral, anti-inflammatory, and antimicrobial activities (Chen et al. 2012; Kumar and Pandey 2013; Favela-Hernández et al. 2016; Mahato et al. 2019; Martinidou et al. 2024; Rodrigues and Pintado 2024).

#### TPC quantification

The quantification of TPC using the *Folin-Ciocalteu* method, recognized for its sensitivity, simplicity, and reproducibility in the analysis of phenolic compounds in complex plant matrices (Pérez et al. 2023), indicated that the TPC in OPEa increased proportionally to the extraction time, being 145.17 ± 0.061, 162.01 ± 0.056 and 178.27 ± 0.045 µg GAE/g OPEa at 20, 40, and 60 min of reaction, respectively (Table 4). The standard curve was prepared using gallic acid (C_7_H_6_O_5_ - GAE) standard solutions in methanol at different concentrations and was described by the equation y = 0.03598x − 0.002311 (R^2^= 0.9990) (Fig. S2).

**Table 4:** Quantification of phenolic compounds in the orange peel aqueous extract (OPEa) using the *Folin-Ciocalteu* method, with extraction times of 20, 40, and 60 min

TPC OPEa (µg GAE / g OPEa)		
20 min	40 min	60 min
145.17 ± 0.061	162.01 ± 0.056	178.27 ± 0.045

Orange peel aqueous extract (OPEa); Total phenolic content (TPC); and gallic acid (C_7_H_6_O_5_ - GAE). Values are expressed as mean ± standard deviation of the mean (M ± SD) from experiments performed in triplicate

The increase in TPC observed is consistent with the presence of polyphenolic compounds identified in OPEa through UHPLC-HRMS/MS analysis, which are recognized for participating in the bioreduction and stabilization of SeNPs through bonding and chelation with selenium ions (Borowska et al. 2025). These compounds stand out due to the presence of the phenol functional group, in which an -OH group linked to an aromatic ring confers the ability to donate electrons and neutralize free radicals. This activity is evidenced in the assay by the reduction of the *Folin-Ciocalteu* reagent, observed by the color transition from green to blue and by the progressive increase in intensity over the reaction time, being confirmed spectrophotometrically by absorbance at ~ 760 nm (Pérez et al. 2023).

Although most studies use organic solvents for the extraction of phenolic compounds due to the generally higher yields obtained (Anagnostopoulou et al. 2006; Oboh and Ademosun 2012; Park et al. 2014; Cendrowski et al. 2024), consistentevidence in the literature demonstrates that water alone is also capable of recovering biologically relevant amounts of these compounds under different extraction conditions. Using water as the sole solvent, polyphenols extraction has shown significant results across different methodologies, from simple to more complex approaches: 51.8 mgGAE/gAE by boiling the peel in alkaline water (Chen et al. 2017); 4.42 and 5.83 mgGAE/gAE by magnetic stirring and ultrasound at 35 °C, respectively (Lachos-Perez et al. 2018); infusion and double-boiler processing of orange peel powder with results comparable to methanolic extracts (Xu et al. 2008); and subcritical water extraction (100–374 °C), yielding 31.70 mgGAE/gAE (Lachos-Perez et al. 2018), 5.53 mgGAE/gAE (Lachos-Perez et al. 2020) and 26.13 mgGAE/gAE (Kalompatsios et al. 2022). Collectively, these findings demonstrate that aqueous extraction of phenolic compounds from OPEa, as applied in this study, is feasible and efficient, aligns with Green Chemistry principles, and enables TPC quantification despite the limited solubility of certain compounds (Polshettiwar and Varma 2010).

#### Antioxidant activity (DPPH assay)

Due to the redox properties of phenolic compounds, they exhibited high antioxidant potential, as evidenced by the DPPH method. The results showed that the elimination of the DPPH free radical by OPEa increased over time, reaching 0.75 and 5.34 µg GAE/g OPEa at 30 and 60 min, respectively (Table 5). The standard curve was prepared using gallic acid (GAE) standard solutions in methanol at different concentrations and was described by the equation y = -0.1746x + 0.4333 (R^2^ = 0.9989) (Fig. S3). The antioxidant activity of OPEa was again associated with the presence of polyphenolic compounds with high antioxidant capacity. This activity was confirmed visually on the microplate by the color transition from violet to light yellow, due to the elimination of the DPPH radical, and spectroscopically by the absorbance at 517 nm, reflecting the action of OPEa as a reducing agent, donor of hydrogen atoms, converting DPPH into DPPH-H hydrazine (Kedare and Singh 2011; Foti 2015).

**Table 5:** Measurement of the antioxidant potential of orange peel aqueous extract (OPEa) using the DPPH radical scavenging method, with extraction times of 30 and 60 min

DPPH (µg GAE / g OPEa)	
30 min	60 min
0.75 ± 0.034	5.34 ± 0.037

Orange peel aqueous extract (OPEa); 2,2-diphenyl-1-picrylhydrazyl (DPPH); and gallic acid (C_7_H_6_O_5_ - GAE). Values are expressed as mean ± standard deviation of the mean (M ± SD) from experiments performed in triplicate

The data from this study confirm the feasibility of aqueous extraction, although the antioxidant capacity obtained is lower than that of extracts using organic solvents. Studies using simple extraction methodologies reveal that: the antioxidant activity of OPEa was 1.07 µmolTE/g when prepared at 35 °C by magnetic stirring and ultrasound (Lachos-Perez et al. 2018); aqueous extractions by infusion and water bath had similar performance to methanolic extracts (Xu et al. 2018); and that Xinhui orange peel exhibited the highest activity among the samples evaluated (Chen et al. 2017). Studies using more complex methodologies, with water in subcritical conditions (150 °C), showed that the antioxidant activity of OPEa varies with flow rate (volume/gram), reaching 10.05–16.26 µmolTE/gOPEa for 10–30 mL/min (Lachos-Perez et al. 2018) and 3.38–5.76 µmolTE/gOPEa for the same flow rates (Lachos-Perez et al. 2020), indicating that activity is not at its maximum at lower flow rates, possibly due to the degradation of antioxidant compounds when the material remains longer in the thermal zone.

### Formation of SeNPs-OPEa

**Fig. 2:**
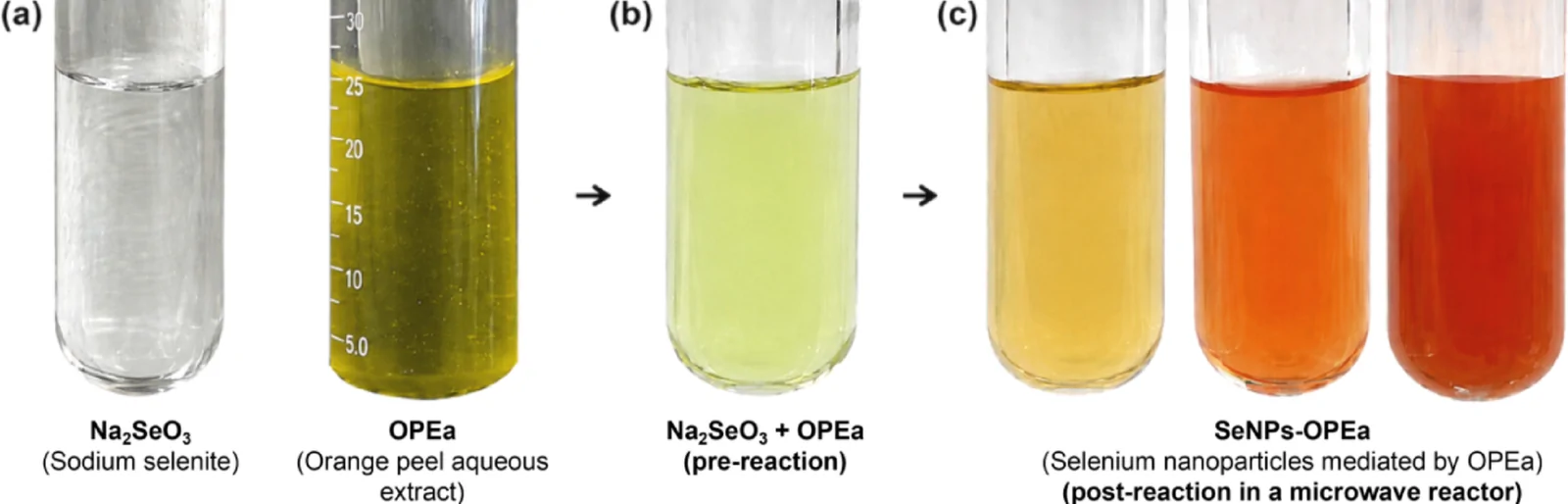
Visual aspect of sodium selenite (Na₂SeO₃) and orange peel aqueous extract (OPEa), and their mixtures, before and after the reaction process, and of the selenium nanoparticles (SeNPs) mediated by OPEa (SeNPs-OPEa). Individual solutions of Na₂SeO₃ and OPEa (**a**); Mixture of Na₂SeO₃ and OPEa before microwave (pre-reaction) (**b**); and SeNPs-OPEa formed after 30 min of microwave reaction at 120–150 °C, with different volumes of OPEa and concentrations of Na₂SeO₃ (post-reaction in a microwave reactor) (**c**)

The use of chromatic characterization of colloids is widely reported in the literature (Aziz et al. 2022; Salem et al. 2022) as preliminary evidence of nanoparticles formation and was applied in this study as an initial criterion to confirm the formation of SeNPs-OPEa (Fig. 2), being subsequently corroborated by spectroscopic and physicochemical analyses. Initially, the Na₂SeO₃ solution was colorless, while the OPEa solution exhibited a yellow-green color (Fig. 2a). When mixed prior to the reaction process, shades ranging from light green to lime green were observed, especially in samples containing higher concentrations of sodium selenite (Na₂SeO₃) (10 mM) and/or higher volumes/concentrations of OPEa (Fig. 2b). After a 30 min reaction in a microwave reactor, the colloidal solutions exhibited color variations depending on the synthesis conditions (Fig. 2c). The samples obtained at 120 °C exhibited shades ranging from light yellow to brownish yellow. The samples synthesized at 150 °C, in turn, exhibited more intense colors, ranging from light yellow to deep red, with the reddest shades observed in the samples with the highest Na₂SeO₃ and/or higher volumes/concentrations of OPEa. A negative control containing only Na₂SeO₃ was subjected to the same synthesis conditions as SeNPs-OPEa, remaining colorless and without detectable changes by Ultraviolet/visible Spectroscopy (UV/Vis) and Dynamic Light Scattering (DLS), indicating that the presence of OPEa is essential for the formation of SeNPs-OPEa.

Red coloration is often reported in biosynthesized SeNPs, regardless of the concentration of selenium precursors, the volumes used, or the type of biological material employed (Gunti et al. 2019; Sheikhlou et al. 2020; Alvi et al. 2021; Hosseini Bafghi et al. 2021; Hosseinpour et al. 2022; Sani-e-Zahra et al. 2022; Hashem et al. 2023; Banerjee & Rajeswari 2024; Martínez-Esquivias et al. 2024; Saranya et al. 2023). Among the few studies reporting the use of orange peel extract in combination with Na₂SeO₃, without or with the use of ascorbic acid, red coloration is also reported (Aziz et al. 2022; Salem et al. 2022). Although red is commonly considered typical of SeNPs, studies indicate that color variations, such as from light yellow to dark red, as observed in the present study, are directly associated with particle size and their spectral and morphological properties (Lin and Wang 2005; Hageman et al. 2017).

**Fig. 3:**
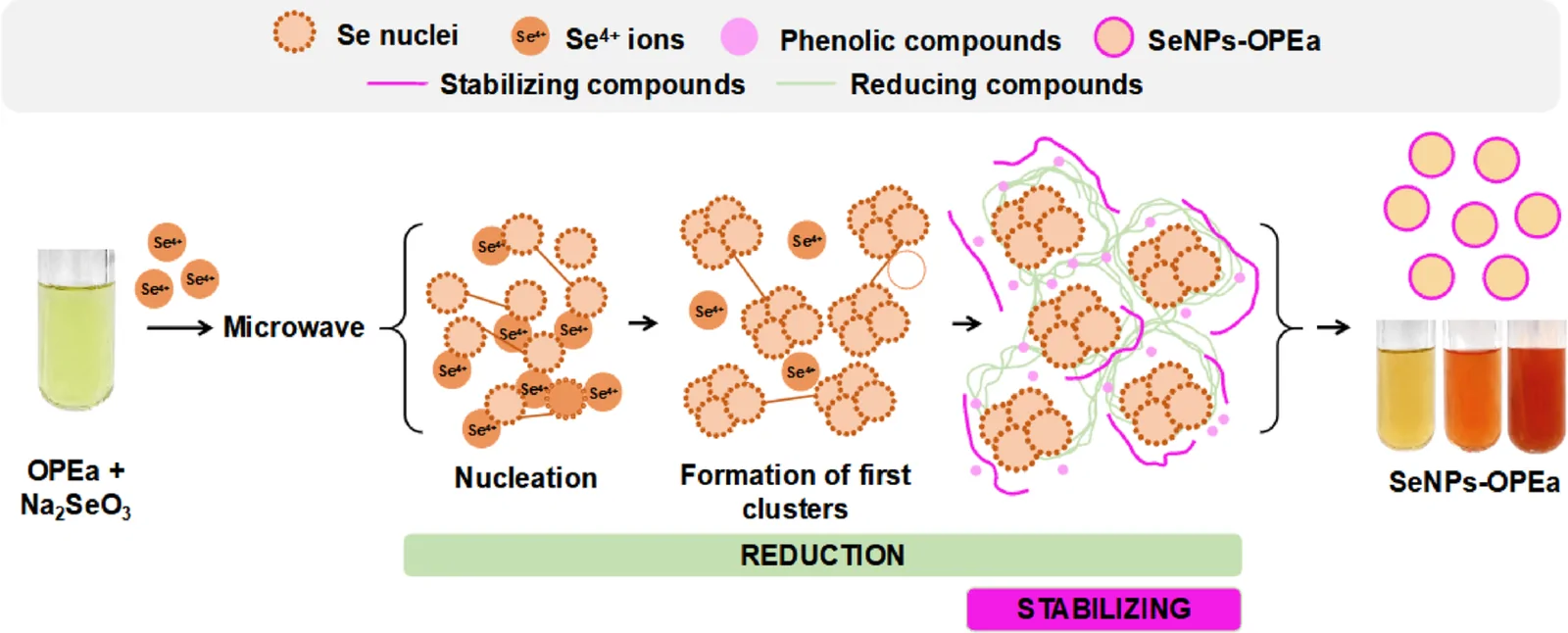
Schematic representation of the possible route for the formation of selenium nanoparticles (SeNPs) mediated by OPEa (SeNPs-OPEa) using orange peel aqueous extract (OPEa) and sodium selenite (Na₂SeO₃)

This initial evaluation showed that the use of a microwave reactor was effective in the formation of SeNPs. In the present study, SeNPs were formed by the reaction between OPEa, composed of polar groups such as hydroxyls and carbonyls, and Na₂SeO₃ dissolved in water, generating sodium ion (Na⁺) and selenite ion ($\:{\mathrm{SeO}}_{\mathrm{3}}^{\mathrm{2-}}$), which enhanced microwave energy absorption through dipolar polarization and ionic conduction (Dallinger and Kappe 2007; Polshettiwar and Varma 2010; Chikan and McLaurin 2016). Theoretically, exposure of the mixture of OPEa and dissociated Na₂SeO₃ ions to the electromagnetic field of the microwave promotes oscillation of dipolar molecules and dielectric heating from dipolar rotation and ionic conduction resulting in homogeneous temperature distribution. These effects can accelerate the reduction of selenium from oxidation state + 4 (Se⁴⁺), present in $\:{\mathrm{SeO}}_{\mathrm{3}}^{\mathrm{2-}}$, to elemental selenium (Se⁰). The reduced selenium atoms subsequently can underwent nucleation and growth, forming Se⁰ nanoparticles. During biosynthesis, phenolic compounds present in the extract can adsorb to the surface of the SeNPs-OPEa, promoting steric and/or electrostatic stabilization, which contributes to reduce nanoparticle aggregation and increasing the colloidal stability of the suspension (Gerbec et al. 2005; Dallinger and Kappe 2007; Nadagouda et al. 2011; Chikan and McLaurin 2016; Ashley et al. 2018) (Fig. 3).

Furthermore, it should be noted that the use of water in all stages of the synthesis was essential, due to its recognized ability to dissipate energy in the form of heat through dielectric heating and dielectric losses. Under high pressure and temperature conditions, such as in a microwave reactor, water can act as a pseudo-organic solvent, with reduced dielectric constant and high ionic product, enhancing the solubility of OPEa biomolecules and facilitating reduction and stabilization reactions, which favor the formation of SeNPs-OPEa (Polshettiwar and Varma 2010).

Previous studies indicate that microwave irradiation is capable of accelerating reactions in solutions containing ions and reducing agents, favoring the formation of homogeneous and stable particles by intensifying dielectric heating and molecular collisions, in addition to reducing reaction times from hours to minutes or seconds, promoting higher yields and cleaner reaction profiles (Kappe 2004; Mellinas et al. 2019; Sheikhlou et al. 2020; Borowska et al. 2023). Moreover, microwave-sealed reactions proved to be preferable because they are scalable and allow for controlled production (Kappe 2004), suggesting that, with further studies on the formation of SeNPs-OPEa, as well as monitoring and optimization, it is possible to obtain stable nanoparticles.

### Characterization of SeNPs-OPEa

The results of characterization by UV/Vis, DLS, Transmission Electron Microscopy (TEM), Scanning electron microscopy (SEM), and energy-dispersive X-ray spectroscopy (EDX) presented below refer to SeNPs-OPEa without purification (WOP). The samples with purification (WP) showed significant changes, including loss of visual characteristics such as decantation and coloration, UV/Vis spectra with broadened bands and absence of defined peaks, as well as abrupt variations in hHydrodynamic diameter (HD), polydispersity index (PdI), and Zeta potential (ZP) values over seven days of analysis. These results indicate that purified samples are unstable in the short term and possibly in the long term. This behavior suggests that this condition may require additional post-synthesis optimization and/or stabilization strategies, including the evaluation of alternative coating agents, different dispersion media, and the adjustment of purification conditions.The results WP are presented in the supplementary material: UV/Vis and visual inspections (Fig. S4) and DLS (HD, PdI and ZP) and pH (Table S5).

#### UV/vis and visual inspection of SeNPs-OPEa

The progress of SeNPs-OPEa synthesis under different conditions was monitored by visual inspection and UV/Vis spectroscopy, a technique recognized for: (1) confirming the formation of nanoparticles based on optical differences in relation to the reagents; (2) monitoring the reaction, characterizing it spectrally and kinetically; and (3) providing crucial insights into the structure, composition, and behavior of nanoparticles (including size, shape, agglomeration, and concentration), which can affect the refractive index near their surface (Barhoum and García-Betancourt 2018).

**Fig. 4:**
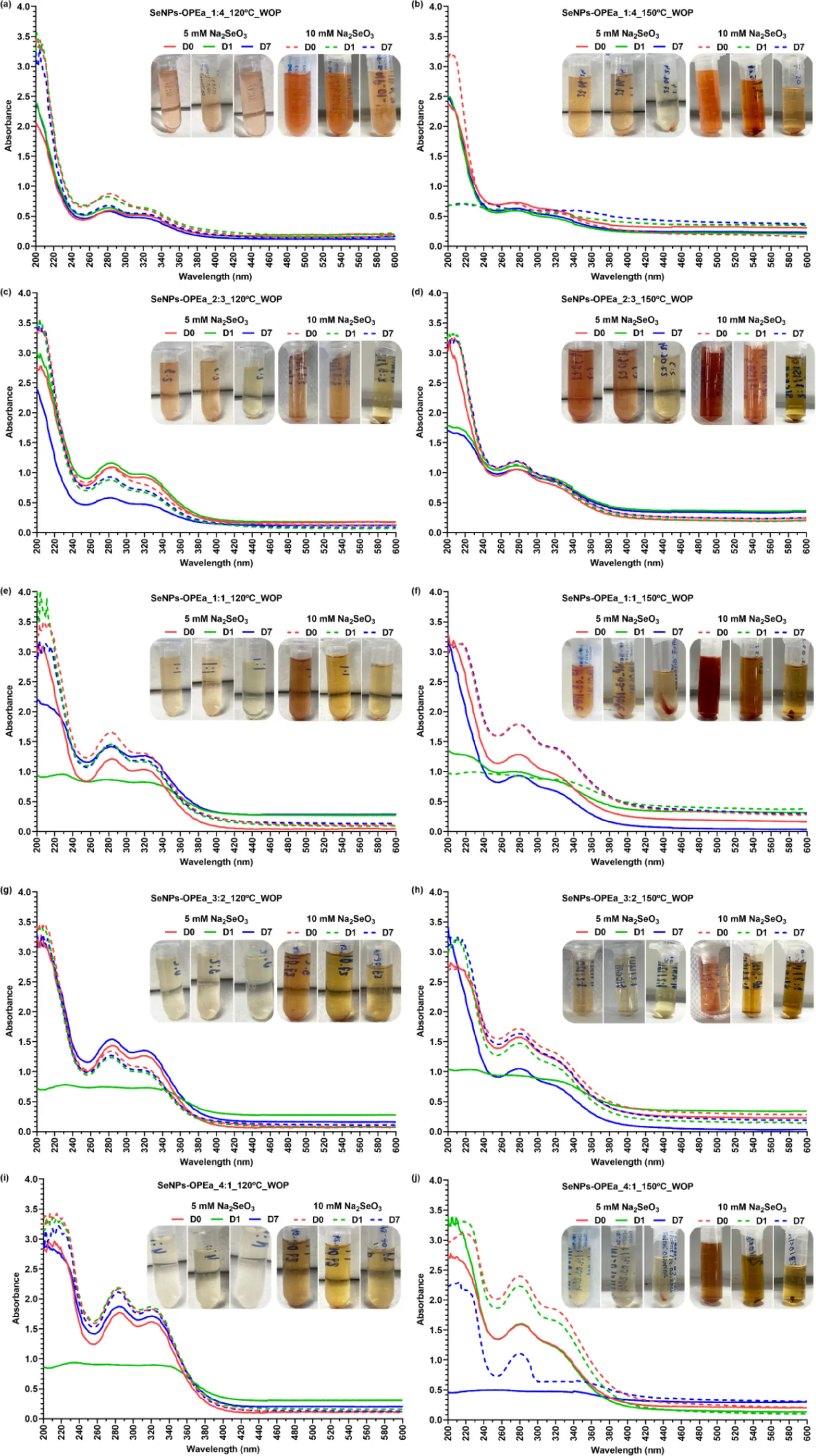
Ultraviolet/Visible Spectroscopy (UV/Vis) absorption spectra of selenium nanoparticles (SeNPs) mediated by orange peel aqueous extract (OPEa) (SeNPs-OPEa), synthesized in a microwave reactor at 120 and 150 °C, without purification (WOP), using sodium selenite (Na₂SeO₃) at concentrations of 5 and 10 mM. The volumetric ratios between OPEa and Na₂SeO₃ (OPEa : Na₂SeO₃) were: 1:4 (3mL:12mL), 2:3 (6mL:9mL), 1:1 (7.5mL:7.5mL), 3:2 (9mL:6mL) and 4:1 (12mL:3mL). The samples were evaluated at different times at room temperature: D0 (day of synthesis), D1 and D7 (subsequent days), with D1 and D7 previously stored in refrigeration (~ 4 °C). The samples are identified as: SeNPs-OPEa_1:4_120ºC_WOP (**a**); SeNPs-OPEa_1:4_150ºC_WOP (**b**); SeNPs-OPEa_2:3_120ºC_WOP (**c**); SeNPs-OPEa_2:3_150ºC_WOP (**d**); SeNPs-OPEa_1:1_120°C_WOP (**e**); SeNPs-OPEa_1:1_150°C_WOP (**f**); SeNPs-OPEa_3:2_120°C_WOP (**g**); SeNPs-OPEa_3:2_150°C_WOP (**h**); SeNPs-OPEa_4:1_120ºC_WOP (**i**); SeNPs-OPEa_4:1_150ºC_WOP (**j**)

The visual inspection over seven days showed that the optical properties varied according to the synthesis conditions, where SeNPs-OPEa synthesized at 120 °C presented a clear, limpid, and translucent coloration in 5 mM Na₂SeO₃, intensifying at 10 mM and partially maintaining colloidal dispersion, while at 150 °C the solutions exhibited more intense coloration and progressive chromatic transitions, such as lightening and sedimentation, regardless of concentration. The UV/Vis analyses confirmed that SeNPs-OPEa synthesized at 120 °C exhibited greater colloidal stability and homogeneous surface plasmon resonance (SPR) bands, while those synthesized at 150 °C exhibited irregular bands, especially with 10 mM Na₂SeO₃. The analyses of SeNPs-OPEa prepared with 5 mM Na₂SeO₃ (solid lines) and 10 mM Na₂SeO₃ (dashed lines) are shown in Fig. 4.

The SeNPs-OPEa_1:4 solutions prepared at 120 °C exhibited nearly overlapping SPR bands regardless of the Na₂SeO₃ concentration, with 10 mM showing higher absorbance intensity but with slight decay, possibly associated with lower colloidal stability, whereas at 150 °C, although the spectral profile remains similar to that at 120 °C, there are signs of lower dispersion stability in macroscopic changes, such as color variation and pellet formation, which are more pronounced at 10 mM, including a reduction in absorbance between D1 and D7 (200–240 nm) relative to D0 (Fig. 4a-b). For SeNPs-OPEa_2:3, SPR band decay, color change, and pellet formation occurred under all conditions except at 150 °C with 10 mM Na₂SeO₃, when the bands remained almost overlapping despite the color change and sedimentation, indicating partial colloidal stability (Fig. 4c-d). For SeNPs-OPEa_1:1, was observed: (1) loss of coloration and pellet formation under all conditions; (2) decay of SPR bands when using 5 mM Na₂SeO₃ (120 and 150 °C) and 10 mM Na₂SeO₃ (150 °C); (3) irregular bands, without a defined peak, with 5 mM Na₂SeO₃ (120 and 150 °C); and (4) only at 120 °C with 10 mM Na₂SeO₃ did more homogeneous bands appear, although still decaying over time (Fig. 4e-f). For SeNPs-OPEa_3:2, it was observed that: (1) at 120 °C, the spectra showed greater homogeneity and probable colloidal stability, while at 150 °C, greater sedimentation and chromatic changes were observed; (2) regardless of temperature, irregular SPR bands were observed when using 5 mM Na₂SeO₃, and more homogeneous bands when using 10 mM (although with visual changes, suggesting probable growth and aggregation of particles) (Fig. 4g-h). For SeNPs-OPEa_4:1: (1) at 120 °C, the colloidal solutions remained stable, without pellet formation, while the opposite occurred at 150 °C; (2) solutions prepared with 5 mM Na₂SeO₃ were clearer and more translucent, and with 10 mM darker and more opaque; (3) decay bands were observed in all conditions tested, except at 120 °C with 10 mM, which remained almost superimposed; and (4) irregular bands, without a defined maximum peak, were observed with 5 mM, regardless of temperature (Fig. 4i-j).

As for the peaks of maximum absorption (λ_max_), sample 1:4 showed a single peak at 282 nm, while 2:3, 1:1, 3:2, and 4:1 showed two peaks (282 and 320 nm). Studies indicate that λ_max_ close to 280 nm indicates the formation of semiconductor SeNPs, whose optical and electronic properties depend on size, allowing light absorption and exciton generation (Borowska et al. 2025). These results suggest that lower volumes of OPEa relative to Na₂SeO₃ produced particles with similar morphologies, while higher OPEa ratios generated particles with distinct morphologies, in agreement with Mie theory and surface plasmon resonance bands (Khodashenas and Ghorbani 2019).The λ_max_ values observed in this study were consistent with the literature: (1) 232–350 nm when the synthesis did not include OPEa (Gunti et al. 2019; Hosseini Bafghi et al. 2021; Hosseinpour et al. 2022; Sani-e-Zahra et al. ; Hashem et al. 2023; Banerjee et al. 2024); (2) 226–500 nm, with up to two λ_max_, when the synthesis included OPEa (Alvi et al. 2021; Salem et al. 2022; Desouky et al. 2025). The SPR bands of the formation reagents differed from those of SeNPs-OPEa, indicating that the synthesis generated nanoparticles with distinct optical-structural properties.

In general, SeNPs-OPEa synthesized at 120 °C presented stable, monodisperse particles and uniform coloration, indicative of homogeneous dispersion, while 150 °C favored aggregation, greater coloration, and pellet formation, especially with 10 mM Na₂SeO₃. Visual inspection over seven days showed that although the absorbance intensity varied, the λ_max_ of the SPR band remained constant, suggesting that the nucleation/maturation process likely continued without significant changes in optical properties. Also, the increase in OPEa concentration under the tested conditions caused an increase in absorbance at 282 nm, accompanied by the appearance of a new peak at 320 nm, similar to that observed in a previous study on the synthesis of SeNPs from orange peel in a microwave oven, using selenium dioxide (SeO₂) and sodium hydroxide (NaOH) (Borowska et al. 2025). Samples with 5 mM Na₂SeO₃, regardless of temperature, remained clear, limpid, and translucent, showing sedimentation only at 150 °C on D7.

#### Analysis by DLS

**Fig. 5:**
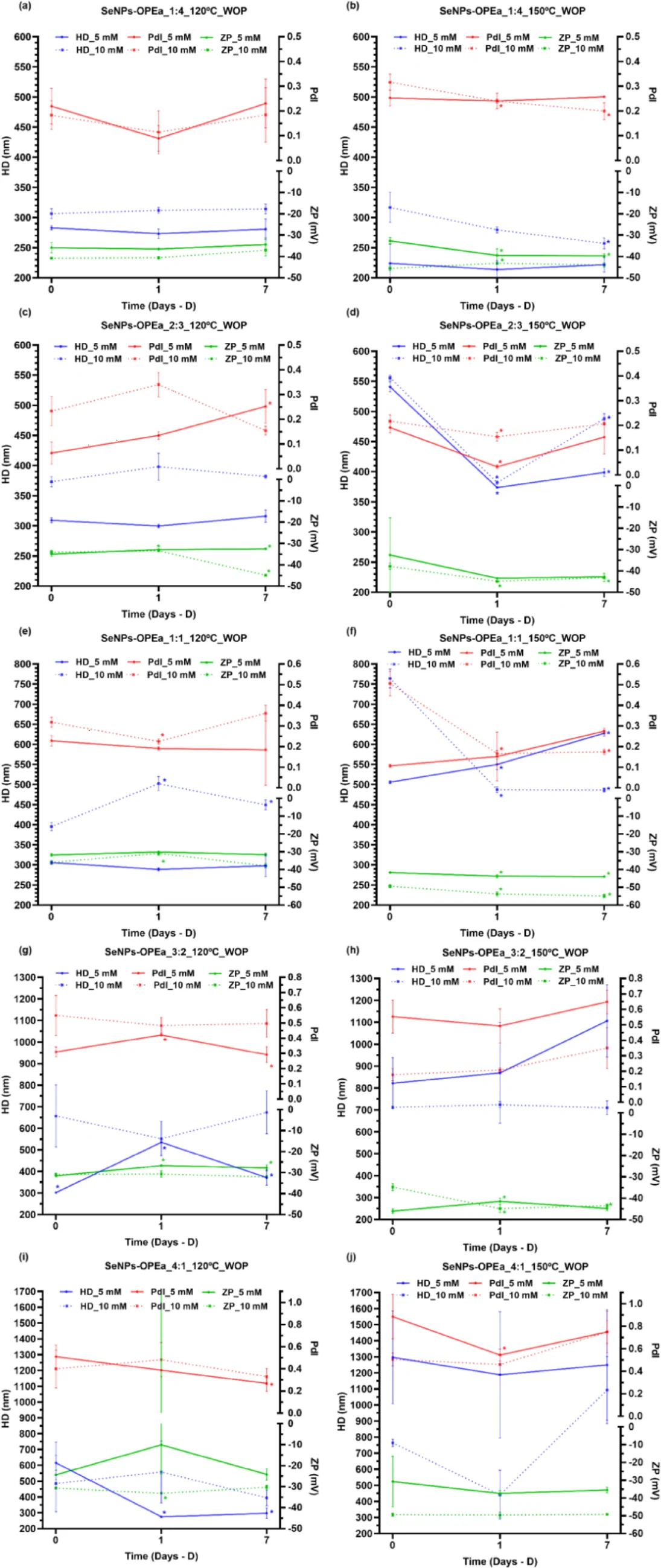
Dynamic light scattering (DLS) analysis of the hydrodynamic diameter (HD), polydispersity index (PdI), and zeta potential (ZP) of selenium nanoparticles (SeNPs) biosynthesized with aqueous orange peel extract (OPEa) (SeNPs-OPEa) in a microwave reactor at 120 and 150 °C, without purification (WOP), using sodium selenite (Na₂SeO₃) at 5 and 10 mM. The volumetric ratios between OPEa and Na₂SeO₃ (OPEa : Na₂SeO₃) were: 1:4 (3 mL:12 mL), 2:3 (6 mL:9 mL), 1:1 (7.5 mL:7.5 mL), 3:2 (9 mL:6 mL), and 4:1 (12 mL:3 mL). Measurements were performed at different times at room temperature: D0 (day of synthesis), D1, and D7 (subsequent days), with D1 and D7 previously stored under refrigeration (4 °C). The symbol ‘*’ indicates statistically significant differences (*p* < 0.05) in relation to D0, evaluated by ANOVA with Tukey’s test, and appears only on days D1 and D7, with blue for HD, red for PdI, and green for ZP. The samples are identified as: SeNPs-OPEa_1:4_120ºC (**a**); SeNPs-OPEa_1:4_150ºC_WOP (**b**); SeNPs-OPEa_2:3_120ºC_WOP (**c**); SeNPs-OPEa_2:3_150ºC_WOP (**d**); SeNPs-OPEa_1:1_120°C_WOP (**e**); SeNPs-OPEa_1:1_150°C_WOP (**f**); SeNPs-OPEa_3:2_120°C_WOP (**g**); SeNPs-OPEa_3:2_150°C_WOP (**h**); SeNPs-OPEa_4:1_120ºC_WOP (**i**); SeNPs-OPEa_4:1_150ºC_WOP (**j**)

The long-term monitoring of the colloidal stability of nanoparticles, assessed by HD, PdI, and ZP, ideally seeks minimal variations over time, since such behavior indicates the formation of colloidal dispersions with smaller particles, homogeneous distribution, high stability, and potential bioavailability (Babick 2020; Kaliva and Vamvakaki 2020; Abdelaziz et al. 2025). The results are shown in Fig. 5, with the mean values detailed in Table S6 (WOP).

In the present study, SeNPs-OPEa showed variations in the mean values of all parameters over time, with only the 1:4 (5 and 10 mM Na₂SeO₃), 1:1 (5 mM Na₂SeO₃) solutions at 120 °C, and 4:1 (10 mM Na₂SeO₃) at 150 °C did not show statistically significant differences (*p* < 0.05) in HD, PdI, and ZP compared to the day of synthesis (D0). Considering the parameters evaluated, HD stood out for its broadened distribution with increased OPEa volume, Na₂SeO₃ concentration, and/or synthesis temperature, possibly due to differential particle growth during the coalescence/aggregation process, as well as precursor consumption in samples with a higher proportion of OPEa relative to Na₂SeO₃. In general, colloidal dispersions with brown or reddish coloration and pellet formation during storage showed greater dimensional variations. As for the PdI, all SeNPs-OPEa samples exhibited values within the range reported in the literature (Kumar and Dixit 2017), with the exception of a few samples that showed values greater than 0.5, indicating increased polydispersity and, consequently, greater heterogeneity of the dispersions at 120 °C (10 mM: 4:1) and at 150 °C (5 mM: 3:2 and 4:1; 10 mM: 4:1). Regarding ZP, all samples exhibited negative surface charges, possibly associated with the presence of OPEa biomolecules adsorbed onto the nanoparticle surface (Abdelaziz et al. 2025). The magnitude of the ZP (|ζ|) was considerably different from zero, indicating colloidal stability. Most samples showed high stability (|ζ| > 30 mV), regardless of temperature, Na₂SeO₃ concentration, or reagent ratio. Exceptions were observed for SeNPs synthesized at a 3:2 and 4:1 ratio, prepared at 120 °C with 5 mM Na₂SeO₃, which exhibited moderate stability (± 20–30 mV) (Clogston and Patri 2010; Kumar and Dixit 2017; Bhattacharjee 2016).

For SeNPs-OPEa_120°C: sample 1:4 stood out for presenting the lowest HD and PdI values and the highest ZP values, regardless of the Na₂SeO₃ concentration used, in comparison with the other samples: 5 mM Na₂SeO₃ (HD: 273.2–282.8 nm; PdI: 0.089–0.231; ZP: -34.4 to -36.5 mV) or 10 mM Na₂SeO₃ (HD: 306.3–314.3 nm; PdI: 0.114–0.184; ZP: -37.1 to -40.8 mV) (Fig. 5a); 2:3, the use of 5 mM Na₂SeO₃ resulted in greater monodispersity, with the lowest PdI (0.064 at D0), while 10 mM Na₂SeO₃ presented higher HD and ZP values (Fig. 5c); for 1:1, the increase in Na₂SeO₃ concentration showed an increase in all parameters when compared to 5 mM (Fig. 5e); for 3:2 was the most unstable sample, showing an increase in HD, PdI, and ZP when the concentration of Na₂SeO₃ was increased, with variations observed on all days of analysis (Fig. 5g); and 4:1 stood out at 5 mM Na₂SeO₃, showing a decrease in HD and PdI and the lowest ZP among the samples, while at 10 mM it showed a reduction in HD with stable PdI and ZP (Fig. 5i).

For SeNPs-OPEa_150°C synthesized with 5 or 10 mM Na₂SeO₃: 1:4 (Fig. 5b) and 2:3 (Fig. 5d) showed lower HD at 5 mM, similar PdI, and higher ZP at 10 mM Na₂SeO₃; for 1:1, the use of 10 mM Na₂SeO₃ stood out in all parameters, presenting lower HD (427.9–487.6 nm) and PdI (0.057–0.174) and higher ZP (-53.0 to -54.9 mV) compared to 5 mM Na₂SeO₃ (Fig. 5f); for 3:2, the lowest HD and PdI were observed at 10 mM, similar to the behavior of 1:1, while the highest ZP occurred at 5 mM (Fig. 5h); and for 4:1, the use of 10 mM presented lower values than 5 mM Na₂SeO₃, although both remained higher than the other samples in all parameters (Fig. 5j).

It should be emphasized that few studies use orange extract, regardless of the part used, in conjunction with Na₂SeO₃, and provide HD, PdI, and ZP data in relation to SeNPs biosynthesis, making accurate comparisons difficult but highlighting the originality of the present study. In similar studies, it was found that SeNPs obtained by magnetic stirring from an aqueous extract of crushed peels coated with chitosan exhibited a smaller HD (58.77 nm) and reduced ZP (-7.94 mV) (Desouky et al. 2025), whereas aqueous SeNPs synthesized exclusively from orange peel extract exhibited a substantially larger HD (756.1 nm) and a ZP closer to neutrality (-14.5 mV) (Borowska et al. 2025).

#### pH analyses

The pH analysis of SeNPs-OPEa was also performed to investigate possible correlations with the results of DLS, visual inspection, and UV/Vis, since variations in this parameter can influence the physicochemical properties (size, uniformity, colloidal stability, and optical properties) of nanoparticles (Al-Gebory and Mengüç 2018). The analyses revealed that the pH did not vary significantly over the days analyzed individually in each sample, with minimal variations observed (Table 6).

**Table 6:** pH of selenium nanoparticles (SeNPs) mediated by orange peel aqueous extract (OPEa) (SeNPs-OPEa), synthesized using sodium selenite (Na_2_SeO_3_) at different reaction temperatures (120 °C and 150 °C) and Na₂SeO₃ concentrations (5 and 10 mM) over 7 days of analysis

Sample	Time	120 °C		150 °C	
		Concentration of Na_2_SeO_3_		Concentration of Na_2_SeO_3_	
		5 mM	10 mM	5 mM	10 mM
1:4	D0	6	8	6	7
	D1	6	8	6	7
	D7	6	7	6	7
2:3	D0	6	7	6	6
	D1	6	7	6	6
	D7	6	7	5	5
1:1	D0	5	6	6	6
	D1	5	6	5	6
	D7	5	6	5	6
3:2	D0	5	6	5	6
	D1	5	6	5	6
	D7	5	5	5	5
4:1	D0	5	6	5	5
	D1	5	6	5	5
	D7	5	6	5	5

Time is indicated in days (D): D0 (day of synthesis), D1 (1 day after synthesis) and D7 (7 days after synthesis). The samples follow the volumetric ratio OPEa_**(mL)**_ : Na₂SeO₃_**(mL)**_. The proportions used were: 1:4 (3 mL : 12 mL), 2:3 (6 mL : 9 mL), 1:1 (7.5 mL : 7.5 mL), 3:2 (9 mL : 6 mL) and 4:1 (12 mL : 3 mL)

The results showed that volumetric ratios with higher OPEa volumes (3:2 and 4:1) had lower pH (5), while those with lower volumes (1:4 and 2:3) maintained a more neutral pH (6–8), suggesting that the excess extract present in the synthesis promoted acidification of the suspension, possibly attributable to the presence of phytochemical compounds in OPEa. The variation in Na₂SeO₃ concentration did not cause significant changes in pH, as evidenced by the samples with higher OPEa volumes (3:2 and 4:1), which remained acidic regardless of the Na₂SeO₃ concentration used. Regardless of the synthesis temperature, samples with lower OPEa volumes (1:4 and 2:3) prepared with 10 mM Na₂SeO₃ had neutral to slightly basic pH (7–8).

#### Influence of microwave and choice of optimized synthesis for new characterizations

Based on the results of UV/Vis, visual inspection, DLS (HD, PdI, and ZP), and pH, microwave reactor-assisted synthesis proved to be fast, simple, energy efficient, and aligned with the principles of green chemistry (Gerbec et al. 2005; Polshettiwar and Varma 2010; Mellinas et al. 2019; Kumar et al. 2020). Considering that biological processes require long reaction times and high temperatures, the microwave reactor proved to be efficient compared to conventional heating methods (Gerbec et al. 2005; Sheikhlou et al. 2020), allowing the formation of SeNPs-OPEa produced under all tested conditions in just 30 min, regardless of the temperature used. The use of microwave reactor irradiation, associated with high temperatures and reagent concentrations/volumes, impacted the stability of the SeNPs-OPEa formed, as reported in previous studies (Kahrilas et al. 2014; Jahan et al.^2021^; Gebishu et al. 2022; Borowska et al. 2025).

The analyses revealed that: (1) The final concentration of the mixture depends on the ratio of OPEa and Na₂SeO₃, with larger volumes of OPEa generating solutions that are more concentrated in extract and diluted in Na₂SeO₃, whereas smaller volumes of OPEa favor the stability of the nanoparticles formed, while larger volumes increase instability; (2) The concentration of Na₂SeO₃ affected all parameters evaluated, with 10 mM favoring rapid nucleation, resulting in aggregation or pellet sedimentation; (3) Temperature significantly influenced the characteristics of the colloids, with 120 °C producing homogeneous, clear, and stable dispersions, even in larger volumes of OPEa (3:2 and 4:1) presenting minor irregularities, and 150 °C resulting in loss of color and pellet sedimentation, regardless of the concentration of Na₂SeO₃; (4) UV/Vis analysis allowed monitoring of nucleation, growth, and colloidal stability by SPR bands, in which irregular or decayed signals indicated aggregation or instability, while homogeneous and almost overlapping signals indicated stable particles, with reagent proportions and temperature significantly influencing the observed patterns; (5) When prepared with 5 mM Na₂SeO₃ at 120 °C, the nanoparticles showed lower HD and PdI, similar ZP, and maintained visual and spectral stability, whereas 10 mM at 120 °C resulted in opposite behavior, and 5 mM at 150 °C generated more heterogeneous and unstable dispersions, evidenced by color variations and pellet formation; (6) The ratio of OPEa to Na₂SeO₃ determined the formation of SeNPs-OPEa, influencing the homogeneity, size, dispersion, and optical properties of the particles.

In view of these observations, the variation in the observed characteristics can be attributed to the synthesis conditions, including the ratio and concentration of the reagents, stirring rate, power, reaction time, and temperature in the microwave reactor. Within the conditions evaluated, synthesis at 120 °C with 5 mM Na₂SeO₃ proved to be the most suitable, producing SeNPs-OPEa with lower HD, clear and translucent colloids, and homogeneous absorbance spectra, representing the ideal combination of stability and quality of the nanoparticles. Considering this, SeNPs-OPE_1:4 synthesized at 120 °C with 5 mM Na₂SeO₃ (SeNPs-OPE_1:4_120ºC_WOP_5mM) were selected for further characterization because they exhibited more homogeneous physicochemical properties, remained stable throughout the analysis period, showed no significant changes compared to the other conditions tested, and did not exhibit statistically significant differences (*p* < 0.05) throughout the analysis period.

#### Morphological and elemental characterization by TEM, SEM, and EDX

**Fig. 6:**
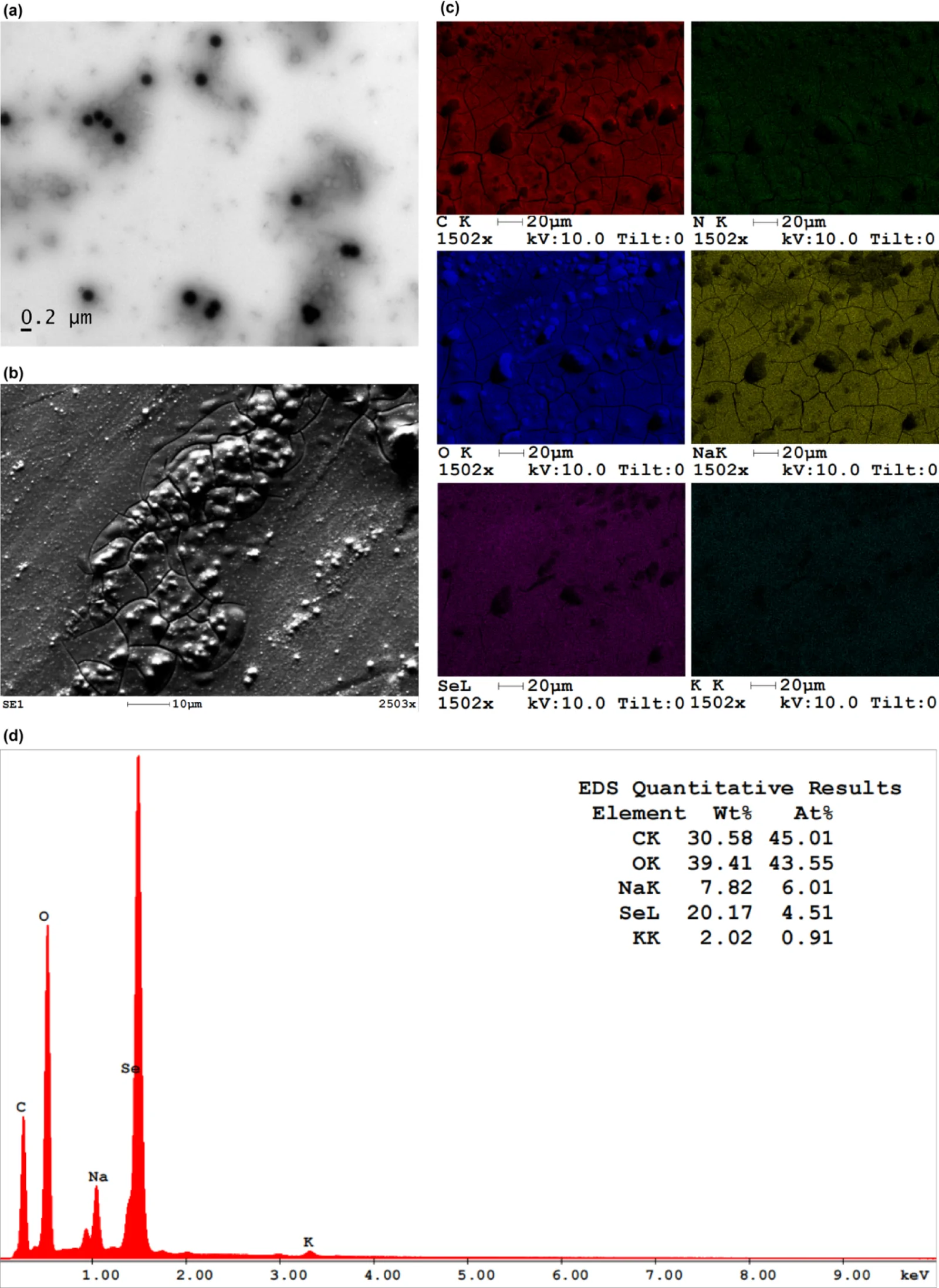
Transmission electron microscopy (TEM), scanning electron microscopy (SEM), energy-dispersive X-ray spectroscopy (EDX), and elemental mapping of selenium nanoparticles (SeNPs) mediated by orange peel aqueous extract (OPEa) (SeNPs-OPEa), synthesized using sodium selenite (Na_2_SeO_3_) at 5 mM. SeNPs-OPEa were prepared in a microwave reactor at a ratio of 1:4 (OPEa_(mL)_: Na₂SeO₃_(mL)_) at 120 °C for 30 min and did not undergo centrifugation - without purification (WOP). Sample name: SeNPs-OPE_1:4_120ºC_WOP_5mM. TEM at 3.000× magnification (**a**); SEM at 2503× magnification (**b**); and Elemental map at 1502x (**c**); EDX (**d**). The elemental map and EDX graph highlight the distribution and composition of the elements present in the SeNPs-OPEa sample

The characterization of morphology by electron microscopy and compositional analysis by EDX was conducted exclusively under the previously selected synthesis condition (SeNPs-OPE_1:4_120ºC_WOP_5mM) (Fig. 6).

The TEM (Fig. 6a) revealed a predominantly spherical morphology with shading around the particles, while the SEM (Fig. 6b) did not allow for detailed visualization of this morphology, but showed a surface layer covering the particles. The layers observed around and on the SeNPs-OPEa are possibly associated with biomolecules derived from OPEa acting as stabilizing agents, as reported in previous studies with SeNPs synthesized from different plant extracts (Dang-Bao et al. 2022). The SeNPs had a dry diameter evaluated by TEM ranging from 58.06 to 71.26 nm. The micrographs showed low nanoparticles density in different regions, as observed in a previous study of SeNPs synthesis in a microwave reactor with orange peel, SeO_2_, and NaOH, suggesting, at first, that the colloidal solution was polydisperse (Borowska et al. 2025). However, quantitative analyses did not confirm this pattern, indicating that associated analysis factors, such as sample preparation for microscopic analysis, may have influenced the observed distribution (Borowska et al. 2025).

Among the few studies describing the biosynthesis of SeNPs from aqueous extracts of orange peel pieces, spherical morphology is reported, in agreement with the findings obtained in the present study. When prepared in a ratio of 1:9 (extract_(mL)_ : Na_2_SeO_3(mL)_): 16–95 nm (200 mg/mL extract and 2 mM Na_2_SeO_3_) (Salem et al. 2022) and 30.2–116 nm (33 mg/mL extract and 0.2 mM Na_2_SeO_3_) (Abdelaziz et al. 2025). When prepared in a 1:1 ratio (extract_(mL)_ : SeO_2(mL)_): 29.24–59.14 nm (30 mg/mL extract 0.05 mM SeO_2_) (Hadi and Mohamed 2025). When prepared in a ratio of 4:1 (extract_(mL)_ : H_2_SeO_3(mL)_): 5–40 nm (50 mg/mL extract 80 mM selenious acid - H_2_SeO_3_) (Dang-Bao et al. 2022). Recent studies have shown that SeNPs synthesized with orange juice or peel extract, using SeO_2_ and NaOH in a microwave reactor, vary in size depending on the extract: 50–135 nm with citrus juice and 70–250 nm with peel, suggesting that the type of extract may influence the size of the nanoparticles (Borowska et al. 2025). Biosynthesis with other biological inputs, such as, for example using *Urtica dioica* (stinging nettle) leaf extract, is also reported for SeNPs with spherical morphology presenting similar average diameters (Hashem and Salem 2022).

The elemental composition of SeNPs-OPE_1:4_120ºC_WOP_5mM was determined by EDX, coupled with a scanning electron microscope, based on the detection of the interaction of characteristic X-rays emitted by the sample. The images in different colors correspond to the mapping of the elements detected in the sample, showing their respective spatial distribution in relation to the spectrum obtained (Fig. 6c). The analyses revealed the presence of selenium (Se) from an absorption signal of approximately 1.5 keV, following patterns reported in the literature (Ho et al. 2021; Shirmehenji et al. 2021; Dang-Bao et al. 2022; Yanez-Lemus et al. 2022), presenting itself as the main constituent of the sample, with a mass proportion of 20.17% (Fig. 6d). The analysis showed the presence of carbon (C), oxygen (O), sodium (Na), and potassium (K), possibly originating from residues of phytochemical compounds in OPEa previously identified by UHPLC-HRMS/MS, which, as observed in TEM and SEM images (Salem et al. 2022), with mass proportions of 30.58%, 39.41%, 7.82%, and 2.02%, respectively (Fig. 6c).

## Conclusions

This study synthesized selenium nanoparticles (SeNPs) based on the principles of sustainability and green chemistry, using water as the solvent throughout all stages and orange peel waste to obtain the aqueous extract (OPEa), which served as a reducing and stabilizing agent. The extract was reacted with sodium selenite (Na₂SeO₃) to form eco-friendly selenium nanoparticles mediated by OPEa (SeNPs-OPEa). We sought to establish a rapid synthesis in a microwave reactor, varying the concentrations and volumes of the reagents (OPEa and Na₂SeO₃) and the synthesis temperature, whose combination of variables tested in this study had not yet been explored in the literature in this way. The characterization of SeNPs-OPEa indicated the formation of particles under all conditions tested, with properties dependent on the ratio and concentration of the reagents, as well as the synthesis temperature. The SeNPs-OPEa with the best characteristics were observed when prepared at 120 °C, with a lower volume of OPEa and a higher volume of Na₂SeO₃ (5 mM), presenting hydrodynamic diameter (HD), polydispersity index (PdI), and Zeta potential (ZP) of up to 316 nm, 0.253, and − 35.9 mV, respectively, ultraviolet/visible spectroscopy (UV/Vis) spectra with homogeneous bands, stable pH, and visual inspections indicating no or minimal pellet formation. In general, SeNPs-OPEa prepared at 150 °C or with higher volumes of Na₂SeO₃ presented colloidal solutions that were more unstable over time, exhibiting clear losses of their characteristics.

Among the conditions tested, the 1:4 condition prepared at 120 °C presented the best DLS characteristics (HD: 273.2–282.8 nm, PdI: 0.089–0.231, ZP: -34.4 to -36.5 mV), UV/Vis (homogeneous bands), translucent visual inspection and no pellet formation, spherical morphology (TEM/SEM) with a dry diameter of 58.06–71.26 nm (TEM) and a high percentage of selenium by EDX (20.17%). These findings reinforce the potential for synthesizing SeNPs from agro-industrial waste, especially from oranges, which has yet to be fully explored, laying the groundwork for future studies focused on optimizing the synthesis, evaluating long-term stability, improving purification and characterization steps, and investigating their biological activities, including potential antioxidant and antimicrobial effects.

## Supplementary Information

Below is the link to the electronic supplementary material.

Supplementary Material 1

## Data Availability

The data presented in this study are available upon request from the authors.
