# Supplementary material for: Biosynthesis of selenium nanoparticles with orange peel waste in microwave: characterization, and spectral properties
Source: 3 Biotech. 2026 Nov 2;16(11):487. doi: 10.1007/s13205-026-05115-0 (PMC13728017; doi:10.1007/s13205-026-05115-0)
Supplement: Supplementary file 1 [file 13205_2026_5115_MOESM1_ESM.docx]

**Supplementary material**

**Table S1** Volumes of reagents used for the *Folin-Ciocalteu* assay in a 96-well microplate with a sample of orange peel aqueous extract (OPEa)

| **Sample** | **Water**  **(mL)** | ***Folin-Ciocalteu* 1 N (mL)** | **Sample**  **(mL)** | **Methanol**  **(mL)** | **Na_2_CO_3_ (mL)** | **V_f_ (mL)** |
| --- | --- | --- | --- | --- | --- | --- |
| Blank* | 0.200 | 0.020 | - | 0.020 | 0.060 | 0.300 |
| Sample-Blank | 0.280 | - | 0.020 | - | - | 0.300 |
| Sample (OPEa) | 0.180 | 0.020 | 0.020 | 0.020 | 0.060 | 0.300 |

Source: Pires et al. (2017a), without adaptations. Na_2_CO_3_ (Sodium carbonate) and V_f_ (Final volume). Note: “*” The absorbance value of the Blank is the same as that obtained for zero concentration of the standard curve

**Table S2** Volumes of reagents used to construct the gallic acid standard curve for 24-well microplates

| **Final concentration in the well (mg/mL)** | **Water**  **(mL)** | ***Folin-Ciocalteu* 1 N (mL)** | **Gallic acid stock solution (200 mg/mL)** | **Methanol**  **(mL)** | **Na_2_CO_3_ (mL)** | **V_f_ (mL)** |
| --- | --- | --- | --- | --- | --- | --- |
| 0^*^ | 0.800 | 0.080 | - | 0.080 | 0.240 | 1.200 |
| 0.002 | 0.800 | 0.080 | 0.012 | 0.068 | 0.240 | 1.200 |
| 0.004 | 0.800 | 0.080 | 0.024 | 0.056 | 0.240 | 1.200 |
| 0.006 | 0.800 | 0.080 | 0.036 | 0.044 | 0.240 | 1.200 |
| 0.008 | 0.800 | 0.080 | 0.048 | 0.032 | 0.240 | 1.200 |
| 0.010 | 0.800 | 0.080 | 0.060 | 0.020 | 0.240 | 1.200 |

Source: Pires et al. (2017a), with adaptations. Na_2_CO_3_ (Sodium carbonate); and V_f_ (Final volume). Note: “*” The absorbance of concentration 0 (Blank) must be discounted in all absorbances of each of the points on the standard curve

**Table S3** Reagent volumes used for the DPPH assay in a 96-well microplate containing an orange peel aqueous extract (OPEa) sample

| **Sample** | **DPPH solution (mL)** | **Sample (mL)** | **Methanol (mL)** | **V_f_ (mL)** |
| --- | --- | --- | --- | --- |
| DPPH Control | 0.280 | - | 0.020 | 0.300 |
| Blank* | - | - | 0.300 | 0.300 |
| Sample-Blank** | - | 0.020 | 0.280 | 0.300 |
| Sample (OPEa) | 0.280 | 0.020 | - | 0.300 |

Source: Pires et al. (2017b), without adaptations. DPPH (2,2-diphenyl-1-picrylhydrazyl); and V_f_ (Final volume). Note: 1) “*” The absorbance value of the Blank must be discounted from the absorbance of the DPPH Control; and 2) “**” The absorbance of the Blank-sample was discounted from the respective sample to correct interferences in the reading

**Table S4 Volumes of reagents used to construct the standard curve for the DPPH assay, using gallic acid for a 24-well microplate**

| **Final concentration in the well (mg/mL)** | **DPPH solution**  **(mL)** | **Gallic acid solution**  **(100 mg/mL)** | **Methanol(mL)** | **V_f_ (mL)** |
| --- | --- | --- | --- | --- |
| Blank* | - | - | 1.200 | 1.200 |
| 0** | 1.120 | - | 0.080 | 1.200 |
| 0.0005 | 1.120 | 0.006 | 0.074 | 1.200 |
| 0.001 | 1.120 | 0.012 | 0.068 | 1.200 |
| 0.0015 | 1.120 | 0.018 | 0.062 | 1.200 |
| 0.002 | 1.120 | 0.024 | 0.056 | 1.200 |

Source: Pires et al. (2017b), with adaptations. DPPH (2,2-diphenyl-1-picrylhydrazyl); and V_f_ (Final volume). Note: 1) “*” Concentration 0 also corresponds to the blank. The absorbance of the blank should be subtracted from all absorbances of each point on the standard curve; and 2) “**” Concentration 0 also corresponds to the DPPH control

**Fig. S1** Ultra-High-Performance Liquid Chromatography coupled to High-Resolution Mass Spectrometry (UHPLC-HRMS/MS) analysis of the orange peel aqueous extract (OPEa), showing the eluted compounds and their respective mass-to-charge ratios (*m/z*) identified from the mass spectra in positive ionization mode (ESI(+)-MS) (a); and negative ionization mode (ESI(-)-MS) (b)

**Fig. S2** Standard curve of gallic acid at concentrations of 0 to 0.010 mg/mL *versus* absorbances read at 760 nm for quantification of total phenols by the *Folin-Ciocalteu* method

**Fig. S3** Standard curve of gallic acid at concentrations of 0 to 0.002 mg/mL *versus* absorbances read at 517 nm to determine the antioxidant capacity against the DPPH radical

**Fig. S4** Ultraviolet/Visible Spectroscopy (UV/Vis) absorption spectra of selenium nanoparticles (SeNPs) mediated by orange peel aqueous extract (OPEa) (SeNPs-OPEa), synthesized in a microwave reactor at 120 and 150 °C, with purification (WP) by centrifugation (32.300 *g*, 15 min, 25 °C), using sodium selenite (Na₂SeO₃) at concentrations of 5 and 10 mM. The volumetric ratios between OPEa and Na₂SeO₃ (OPEa_(mL)_ : Na₂SeO₃_(mL)_) were: 1:4 (3mL:12mL), 2:3 (6mL:9mL), 1:1 (7.5mL:7.5mL), 3:2 (9mL:6mL) and 4:1 (12mL:3mL). The samples were evaluated at different times at room temperature: D0 (day of synthesis), D1 and D7 (subsequent days), with D1 and D7 previously stored in refrigeration (~4°C). **The samples are identified as: SeNPs-OPEa_1:4_120ºC_WP (a); SeNPs-OPEa_1:4_150ºC_WP (b); SeNPs-OPEa_2:3_120ºC_WP (c); SeNPs-OPEa_2:3_150ºC_WP (d); SeNPs-OPEa_1:1_120°C_WP (e); SeNPs-OPEa_1:1_150°C_WP (f); SeNPs-OPEa_3:2_120°C_WP (g); SeNPs-OPEa_3:2_150°C_WP (h); SeNPs-OPEa_4:1_120ºC_WP (i); SeNPs-OPEa_4:1_150ºC_WP (j);**

**Table S5** Dynamic light scattering (DLS) analysis of the hydrodynamic diameter (HD), polydispersity index (PdI), and zeta potential (ZP) of selenium nanoparticles (SeNPs) mediated by aqueous orange peel extract (OPEa) (SeNPs-OPEa), with purification (WP)

|  | | **120 ºC** | | | | | | | |
| --- | --- | --- | --- | --- | --- | --- | --- | --- | --- |
|  |  | **5 mM (Na_2_SeO_3_)** | | | | **10 mM (Na_2_SeO_3_)** | | | |
| **Sample** | **Time** | **HD (nm)** | **PdI** | **ZP (mV)** | **pH** | **HD (nm)** | **PdI** | **ZP (mV)** | **pH** |
| **1:4** | **D0** | 285.9 ± 2.67 | 0.223 ± 0.03 | -43.1 ± 0.76 | 5 | 313.8 ± 5.20 | 0.163 ± 0.02 | -35.4 ± 0.85 | 5 |
|  | **D1** | 294.4 ± 2.34 | 0.193 ± 0.02 | -44.8 ± 0.67 | 5 | 322.5 ± 2.75^*^ | 0.261 ± 0.06 | -29.8 ± 0.49^*^ | 5 |
|  | **D7** | 276.4 ± 5.33^*^ | 0.164 ± 0.07 | -38.9 ± 0.57^*^ | 5 | 325.8 ± 1.15^*^ | 0.142 ± 0.07 | -22.5 ± 0.51^*^ | 5 |
| **2:3** | **D0** | 337.3 ± 35.30 | 0.288 ± 0.08 | -38.4 ± 1.31 | 5 | 373.8 ± 14.55 | 0.221 ± 0.02 | -34.7 ± 1.72 | 7 |
|  | **D1** | 313.2 ± 12.48 | 0.207 ± 0.07 | -40.2 ± 0.51 | 5 | 423.3 ± 19.65 | 0.361 ± 0.05^*^ | -38.4 ± 0.55 | 7 |
|  | **D7** | 318.6 ± 15.41 | 0.243 ± 0.04 | -39.0 ± 0.23 | 5 | 418.1 ± 30.35 | 0.351 ± 0.05^*^ | -40.0 ± 1.88^*^ | 5 |
| **1:1** | **D0** | 459.4 ± 116.60 | 0.419 ± 0.09 | -36.5 ± 2.90 | 5 | 399.4 ± 7.67 | 0.123 ± 0.08 | -32.6 ± 0.20 | 6 |
|  | **D1** | 405.2 ± 11.95 | 0.299 ± 0.06 | -34.7 ± 3.95 | 5 | 426.0 ± 18.11 | 0.289 ± 0.11 | -33.3 ± 0.15 | 6 |
|  | **D7** | 296.9 ± 23.96 | 0.245 ± 0.20 | -40.7 ± 0.20 | 5 | 430.4 ± 3.71^*^ | 0.286 ± 0.07 | -34.3 ± 2.16 | 6 |
| **3:2** | **D0** | 469.5 ± 26.93 | 0.314 ± 0.04 | -40.5 ± 1.32 | 5 | 805.0 ± 431.40 | 0.649 ± 0.32 | -37.2 ± 3.06 | 6 |
|  | **D1** | 372.2 ± 50.25 | 0.342 ± 0.09 | -32.9 ± 2.14^*^ | 5 | 978.6 ± 380.20 | 0.808 ± 0.24 | -33.4 ± 1.97 | 6 |
|  | **D7** | 372.2 ± 29.13 | 0.333 ± 0.07 | -35.5 ± 4.13 | 5 | 1216 ± 644.80 | 0.801 ± 0.20 | -35.2 ± 2.06 | 5 |
| **4:1** | **D0** | 1872 ± 1448 | 0.834 ± 0.17 | -36.3 ± 6.12 | 5 | 534.8 ± 280.20 | 0.428 ± 0.31 | -21.9 ± 1.83 | 6 |
|  | **D1** | 1590 ± 619.00 | 1.000 ± 0.00 | -26.5 ± 0.25^♦^ | 5 | 701.4 ± 261.20 | 0.585 ± 0.22 | -32.1 ± 0.93^*^ | 6 |
|  | **D7** | 650.7 ± 31.50 | 0.496 ± 0.03^*^ | -30.1 ± 3.63 | 5 | 598.7 ± 263.30 | 0.397 ± 0.34 | -30.4 ± 1.73^*^ | 6 |
|  | | **150 ºC** | | | | | | | |
|  |  | **5 mM (Na_2_SeO_3_)** | | | | **10 mM (Na_2_SeO_3_)** | | | |
| **Sample** | **Time** | **HD (nm)** | **PdI** | **ZP (mV)** | **pH** | **HD (nm)** | **PdI** | **ZP (mV)** | **pH** |
| **1:4** | **D0** | 213.3 ± 4.16 | 0.232 ± 0.05 | -25.5 ± 0.50 | 5 | 661.7 ± 64.75 | 0.596 ± 0.08 | -38.8 ± 0.31 | 6 |
|  | **D1** | 215.2 ± 8.41 | 0.241 ± 0.04 | -50.5 ± 0.70^*^ | 5 | 455.4 ± 85.83 | 0.405 ± 0.05 | -39.2 ± 0.21 | 6 |
|  | **D7** | 259.0 ± 10.36^*^ | 0.372 ± 0.05^*^ | -40.0 ± 1.66^*^ | 5 | 623.8 ± 134.50 | 0.535 ± 0.12 | -40.7 ± 0.45^*^ | 6 |
| **2:3** | **D0** | 387.0 ± 8.67 | 0.049 ± 0.04 | -32.5 ± 0.40 | 5 | 589.5 ± 8.57 | 0.165 ± 0.04 | -43.6 ± 0.64 | 6 |
|  | **D1** | 386.1 ± 1.65 | 0.058 ± 0.01 | 24.9 ± 0.90^*^ | 5 | 555.9 ± 1.39^*^ | 0.172 ± 0.04 | -44.5 ± 0.70 | 6 |
|  | **D7** | 392.3 ± 5.45 | 0.157 ±0.03^*^ | -42.5 ± 0.20^*^ | 5 | 373.6 ± 2.98^*^ | 0.169 ± 0.04 | -38.0 ± 0.31^*^ | 5 |
| **1:1** | **D0** | 539.7 ± 6.84 | 0.225 ± 0.07 | -39.7 ± 0.74 | 5 | 436.9 ± 7.91 | 0.073 ± 0.10 | -44.6 ± 1.36 | 5 |
|  | **D1** | 539.8 ± 10.94 | 0.086 ± 0.06^*^ | -44.3 ± 2.76^*^ | 5 | 425.2 ± 1.97 | 0.065 ± 0.09 | -43.4 ± 1.15 | 5 |
|  | **D7** | 550.6 ± 7.96 | 0.136 ± 0.02 | -36.7 ± 1.21 | 5 | 433.8 ± 8.20 | 0.193 ± 0.03 | -43.5 ± 0.31 | 6 |
| **3:2** | **D0** | 838.1 ± 87.67 | 0.425 ± 0.06 | -37.8 ± 1.05 | 5 | 675.4 ± 8.89 | 0.192 ± 0.09 | -44.8 ± 1.48 | 6 |
|  | **D1** | 992.1 ± 283.10 | 0.575 ± 0.25 | -50.2 ± 0.47^*^ | 5 | 672.9 ± 27.78 | 0.521 ± 0.11^*^ | -32.5 ± 0.80^*^ | 6 |
|  | **D7** | 946.8 ± 81.28 | 0.521 ± 0.06 | 29.0 ± 5.01^*^ | 5 | 735.6 ± 32.67 | 0.368 ± 0.12 | -42.7 ± 1.32 | 5 |
| **4:1** | **D0** | 1717 ± 468.60 | 0.898 ± 0.18 | -47.8 ± 1.34 | 5 | 947.5 ± 58.72 | 0.579 ± 0.13 | -37.4 ± 0.85 | 5 |
|  | **D1** | 1107 ± 146.20 | 0.531 ± 0.13 | -43.6 ± 2.78 | 5 | 881.5 ± 33.75 | 0.424 ± 0.13 | -44.6 ± 1.33^*^ | 5 |
|  | **D7** | 1199 ± 107.10 | 0.469 ± 0.18^*^ | -43.0 ± 0.46^*^ | 5 | 1033 ± 117.50 | 0.717 ± 0.13 | -40.2 ± 0.40^*^ | 5 |

HD (Hydrodynamic diameter); PdI (Polydispersity index); and ZP (Zeta Potential). HD, PdI, and ZP values are expressed as mean ± standard deviation of the mean (M ± SD) from measurements performed in triplicate. Statistical analyses included one-way ANOVA (p<0.05) and Tukey's test. Significant differences in the analyzed parameters were compared with measurements performed on the day of synthesis (D0). The symbols used for representation were: ‘*’. **The colloidal stability of the samples was evaluated over seven days, with measurements taken at the following intervals: D0 (day of synthesis), D1, and D7 (subsequent days). All analyses were conducted at room temperature, including those referring to previously refrigerated samples.** Note: SeNPs-OPEa were produced from the synthesis with different concentrations of sodium selenite (Na_2_SeO_3_) (5 and 10 mM) at 120 and 150 ºC, in different volumetric ratios between OPEa_(mL)_: Na₂SeO₃_(mL)_ (1:4, 2:3, 1:1, 3:2 and 4:1). The proportions used were: 1:4 (3 mL : 12 mL), 2:3 (6 mL : 9 mL), 1:1 (7.5 mL : 7.5 mL), 3:2 (9 mL : 6 mL) and 4:1 (12 mL : 3 mL).

**Table S6** Dynamic light scattering (DLS) analysis of the hydrodynamic diameter (HD), polydispersity index (PdI), and zeta potential (ZP) of selenium nanoparticles (SeNPs) mediated by aqueous orange peel extract (OPEa) (SeNPs-OPEa), without purification (WOP)

|  | | | | **120 ºC** | | | | | | | | | | |
| --- | --- | --- | --- | --- | --- | --- | --- | --- | --- | --- | --- | --- | --- | --- |
|  | | | | **5 mM (Na_2_SeO_3_)** | | | | | | **10 mM (Na_2_SeO_3_)** | | | | |
| **Sample** | | **Time** | | **HD (nm)** | | **PdI** | | **ZP (mV)** | | **HD (nm)** | | **PdI** | | **ZP (mV)** |
| **1:4** | | **D0** | | 282.8 ± 3.65 | | 0.219 ± 0.07 | | -35.9 ± 2.48 | | 306,3 ± 8,30 | | 0,183 ± 0,06 | | -40,8 ± 0,55 |
|  |  | **D1** | | 273.2 ± 7.71 | | 0.089 ± 0.05 | | -36.5 ± 0.46 | | 311,8 ± 5,03 | | 0,114 ± 0,09 | | -40,6 ± 0,62 |
|  |  | **D7** | | 280.6 ± 17.27 | | 0.231 ± 0.10 | | -34.4 ± 3.08 | | 314,3 ± 8,18 | | 0,184 ± 0,11 | | -37,1 ± 2,83 |
| **2:3** | | **D0** | | 309.3 ± 4.16 | | 0.064 ± 0.05 | | -35.0 ± 1.11 | | 373,3 ± 8,32 | | 0,233 ± 0,06 | | -34,1 ± 0,76 |
|  |  | **D1** | | 299.6 ± 2.72 | | 0.135 ± 0.02 | | -32.9 ± 0.06^*^ | | 398,2 ± 22,56 | | 0,340 ± 0,05 | | -33,4 ± 0,15 |
|  |  | **D7** | | 316.0 ± 10.40 | | 0.253 ± 0.07^*^ | | -32.5 ± 0.55^*^ | | 406,1 ± 9,58 | | 0,290 ± 0,09 | | -34,6 ± 0,95^*^ |
| **1:1** | | **D0** | | 305.7 ± 4.96 | | 0.228 ± 0.03 | | -31.8 ± 0.95 | | 395.3 ± 10.10 | | 0.318 ± 0.02 | | -36.0 ± 0.78 |
|  |  | **D1** | | 288.5 ± 3.98 | | 0.190 ± 0.01 | | -30.1 ± 0.57 | | 502.0 ± 17.64^*^ | | 0.225 ± 0.01^*^ | | -30.9 ± 1.01^*^ |
|  |  | **D7** | | 297.9 ± 27.19 | | 0.184 ± 0.17 | | -31.6 ± 0.92 | | 449.4 ± 12.60^*^ | | 0.361 ± 0.04 | | -38.0 ± 1.20 |
| **3:2** | | **D0** | | 301.7 ± 4.80 | | 0.310 ± 0.03 | | -31.6 ± 0.35 | | 657.8 ± 144.70 | | 0.549 ± 0.13 | | -30.9 ± 0.35 |
|  |  | **D1** | | 534.5 ± 119.6^*^ | | 0.421 ± 0.10^*^ | | -26.7 ± 2.10^*^ | | 552.0 ± 79.66 | | 0.483 ± 0.05 | | -30.7 ± 1.48 |
|  |  | **D7** | | 371.3 ± 34.98^*^ | | 0.292 ± 0.05 | | -27.8 ± 1.46^*^ | | 590.4 ± 126.10 | | 0.497 ± 0.09 | | -32.0 ± 0.83 |
| **4:1** | | **D0** | | 616.6 ± 131.40 | | 0.510 ± 0.10 | | -24.4 ± 2.17 | | 485.3 ± 179.70 | | 0.399 ± 0.17 | | -30.7 ± 0.50 |
|  |  | **D1** | | 274.4 ± 5.30^*^ | | 0.387 ± 0.07 | | -26.3 ± 2.21 | | 557.7 ± 196.20 | | 0.482 ± 0.15 | | -33.2± 0.49^*^ |
|  |  | **D7** | | 297.0 ± 31.93^*^ | | 0.270 ± 31.93^*^ | | -24.2 ± 2.76 | | 394.1 ± 49.85 | | 0.330 ± 0.07 | | -30.3 ± 0.90 |
|  | | | **150 ºC** | | | | | | | | | | | |
|  | | | **5 mM (Na_2_SeO_3_)** | | | | | | **10 mM (Na_2_SeO_3_)** | | | | | |
| **Sample** | **Time** | | **HD (nm)** | | **PdI** | | **ZP (mV)** | | **HD (nm)** | | **PdI** | | **ZP (mV)** | |
| **1:4** | **D0** | | 223.9 ± 31.43 | | 0.253 ± 0.03 | | -32.7 ± 1.46 | | 316.7 ± 24.83 | | 0.316 ± 0.03 | | -45.6 ± 1.05 | |
|  | **D1** | | 213.6 ± 15.04 | | 0.240 ± 0.03 | | -39.5 ± 3.14^*^ | | 279.5 ± 4.90 | | 0.240 ± 0.01^*^ | | -43.2 ± 0.59^*^ | |
|  | **D7** | | 221.7 ± 12.53 | | 0.257 ± 0.01 | | -39.7 ± 0.96^*^ | | 257.1 ± 9.07^*^ | | 0.200 ± 0.03^*^ | | -44.0 ± 1.04 | |
| **2:3** | **D0** | | 540.9 ± 8.64 | | 0.191 ± 0.02 | | -42.5 ± 0.61 | | 556.0 ± 3.85 | | 0.218 ± 0.03 | | -37.9 ± 1.42 | |
|  | **D1** | | 373.8 ± 1.61^*^ | | 0.033 ± 0.01^*^ | | -43.4 ± 0.49 | | 381.9 ± 3.00^*^ | | 0.154 ± 0.02^*^ | | -44.9 ± 0.30^*^ | |
|  | **D7** | | 398.8 ± 6.41^*^ | | 0.153 ± 0.07 | | -42.8 ± 0.35 | | 488.1 ± 8.52^*^ | | 0.207 ± 0.01 | | -43.2 ± 1.97^*^ | |
| **1:1** | **D0** | | 505.8 ± 3.38 | | 0.106 ± 0.01 | | -41,7 ± 0.31 | | 427.9 ± 4.00 | | 0.057 ± 0.03 | | -53.0 ± 0.62 | |
|  | **D1** | | 550.0 ± 13.20^*^ | | 0.152 ± 0.12^*^ | | -43.8 ± 0.95^*^ | | 487.3 ± 7.35^*^ | | 0.166 ± 0.02^*^ | | -53.8 ± 1.29^*^ | |
|  | **D7** | | 627.7 ± 7.12^*^ | | 0.274 ± 0.01 | | -44.0 ± 0.64^*^ | | 486.6 ± 4.60^*^ | | 0.174 ± 0.01^*^ | | -54.9 ± 1.00^*^ | |
| **3:2** | **D0** | | 821.8 ± 117.10 | | 0.553 ± 0.10 | | -46.1 ± 1.10 | | 712.2 ± 3.12 | | 0.177 ± 0.04 | | -34.8 ± 1.63 | |
|  | **D1** | | 869.0 ± 229.90 | | 0.493 ± 0.11 | | -41.5 ± 1.54^*^ | | 723.8 ± 13.39 | | 0.208 ± 0.01 | | -44.9 ± 1.84^*^ | |
|  | **D7** | | 1107 ± 165.60 | | 0.649 ± 0.08 | | -44.9 ± 1.07 | | 710.0 ± 31.25 | | 0.350 ± 0.13 | | -43.5 ± 0.87^*^ | |
| **4:1** | **D0** | | 1296 ± 288.70 | | 0.885 ± 0.20 | | -30.7 ± 14.42 | | 764.0 ± 23.340 | | 0.506 ± 0.06 | | -49.4 ± 0.90 | |
|  | **D1** | | 1188 ± 393.90 | | 0.544 ± 0.05^*^ | | -37.4 ± 1.55 | | 441.1 ± 153.30 | | 0.460 ± 0.09 | | -49.6 ± 1.61 | |
|  | **D7** | | 1250 ± 344.50 | | 0.750 ± 0.10 | | -35.5 ± 1.55 | | 1093 ± 209.30 | | 0.750 ± 0.18 | | -49.2 ± 0.45 | |

HD (Hydrodynamic diameter); PdI (Polydispersity index); and ZP (Zeta Potential). HD, PdI, and ZP values are expressed as mean ± standard deviation of the mean (M ± SD) from measurements performed in triplicate. Statistical analyses included one-way ANOVA (p<0.05) and Tukey's test. Significant differences in the analyzed parameters were compared with measurements performed on the day of synthesis (D0). The symbols used for representation were: ‘*’. **The colloidal stability of the samples was evaluated over seven days, with measurements taken at the following intervals: D0 (day of synthesis), D1, and D7 (subsequent days). All analyses were conducted at room temperature, including those referring to previously refrigerated samples.** Note: SeNPs-OPEa were produced from the synthesis with different concentrations of sodium selenite (Na_2_SeO_3_) (5 and 10 mM) at 120 and 150 ºC, in different volumetric ratios between OPEa_(mL)_: Na₂SeO₃_(mL)_ (1:4, 2:3, 1:1, 3:2 and 4:1). The proportions used were: 1:4 (3 mL : 12 mL), 2:3 (6 mL : 9 mL), 1:1 (7.5 mL : 7.5 mL), 3:2 (9 mL : 6 mL) and 4:1 (12 mL : 3 mL)
